# A multidimensional measure of pre-service teachers’ perceived technological competence for teaching: integrating TPACK, DigCompEdu, and self-efficacy

**DOI:** 10.3389/fpsyg.2026.1914635

**Published:** 2026-09-08

**Authors:** Ismet Kaya, Rusen Meylani

**Affiliations:** Department of Educational Sciences, Ziya Gökalp Faculty of Education, Dicle University, Diyarbakır, Türkiye

**Keywords:** DigCompEdu, scale validation, self-efficacy, teacher education, technological competence, TPACK

## Abstract

**Background:**

Technological competence for teaching is multidimensional, encompassing digital tool use, professional digital practice, assessment, pedagogical integration, adaptability, and technology-related self-efficacy. Existing measures often assess these components separately rather than within a unified instrument.

**Objective:**

This study developed and validated a multidimensional self-report scale of pre-service teachers’ technological competence for teaching by integrating complementary elements of the Technological Pedagogical Content Knowledge (TPACK) framework, the European Framework for the Digital Competence of Educators (DigCompEdu), and technology-related self-efficacy.

**Methods:**

A multi-phase scale-development and validation design involved two independent cohorts of pre-service teachers at a public university in Türkiye. The pilot sample (*n* = 513) was used for item reduction and exploratory factor analysis (EFA), while the main sample (*n* = 974) was used for confirmatory factor analysis (CFA), reliability and construct-validity analyses, gender measurement-invariance testing, and concurrent associations with a scenario-based technological-pedagogical judgment measure. An initial 90-item pool was reduced to 48 after content review and to 33 following iterative EFA. Principal axis factoring with Promax rotation and diagonally weighted least squares CFA were used.

**Results:**

EFA supported a six-factor, 33-item structure: Foundational Digital Tool Use; Professional Digital Communication and Collaboration; Assessment, Feedback, and Learning Analytics; Pedagogically Aligned Technology Integration; Instructional Technology Troubleshooting and Adaptability; and Teaching-Related Technology Self-Efficacy. The factors explained 62.8% of the variance. CFA showed excellent fit, *χ*^2^(480) = 807.32, CFI = 0.980, TLI = 0.978, RMSEA = 0.026, SRMR = 0.036. AVE ranged from 0.520 to 0.613, HTMT from 0.024 to 0.310, and subscale reliability from *ω* = 0.804 to 0.895. Configural, loading, and threshold invariance were supported across gender. All dimensions correlated positively with scenario-based judgment (rs = 0.218–0.317, ps < 0.001), jointly explaining 35.4% of quiz-score variance.

**Conclusion:**

Findings provide initial psychometric support for a six-dimensional self-report measure of technological competence for teaching. The instrument is best interpreted at the subscale level, and its scores represent self-reported technological competence rather than direct observation of teaching performance. It is appropriate for research, formative use, and program evaluation. Further validation across institutions, longitudinal designs, and independent performance-based measures is recommended.

## 1 Introduction

### 1.1 Overview

Digital technologies now form a routine part of teaching. Online and distance education, the instructional shifts that followed COVID-19, and the wider use of digital platforms have all strengthened expectations that teachers integrate technology into teaching rather than simply operate tools (Huang et al., 2025; Nepembe and Simuja, 2023; Wardani et al., 2020). Studies across educational settings describe this integration through tools such as Google Classroom, presentation software, and other platform-based applications used to support instruction, communication, and learning management (Gromik et al., 2024; Imania et al., 2022). Research also shows that teachers’ technology use relates closely to their judgments about instructional usefulness and to institutional conditions that shape day-to-day practice (Ifinedo et al., 2020; Wu et al., 2015).

Teacher education therefore holds a central role in preparing pre-service teachers for technology-enhanced teaching. Existing research repeatedly identifies a gap between exposure to theoretical coursework and the practical enactment of integrated technology use in authentic teaching contexts (Abubakir and Alshaboul, 2023; Choi and Chung, 2025; Nepembe and Simuja, 2023). At the same time, intervention and coursework studies report growth in pre-service teachers’ TPACK-related knowledge following structured preparation, showing that technology integration develops through deliberate training rather than incidental experience (Bakar et al., 2020; Dağgöl, 2022; Tan et al., 2023).

This issue becomes especially important for pre-service teachers because early professional experiences shape both competence and confidence. Evidence from practicum and online teaching contexts shows that pre-service teachers often rely on basic digital tools and display weaknesses in technology-supported assessment and deeper instructional design, despite generally positive attitudes toward technology use (Abubakir and Alshaboul, 2023; Choi and Chung, 2025). As a result, technological competence for teaching extends beyond general digital skill. It refers to the ability to combine technology, pedagogy, and content in ways that support teaching decisions in real contexts (Ifinedo et al., 2020; Wu et al., 2015).

For this reason, the measurement of pre-service teachers’ technological competence remains a major research concern. Instrument-based studies provide useful domain-specific information by identifying areas that may be comparatively less developed, especially technology-related and integrated domains that connect pedagogy, content, and technology (Hsu et al., 2023; Nurwahidah et al., 2023; Uygun et al., 2023). Addressing this measurement problem requires a theoretical framework that captures not only knowledge for technology integration, but also broader professional digital competence and beliefs about one’s capability to enact technology-supported teaching. The following section therefore brings together TPACK, DigCompEdu, and self-efficacy as complementary foundations for the proposed multidimensional construct.

### 1.2 Theoretical framework

The main theoretical basis of this study is the TPACK framework. TPACK conceptualizes effective technology integration through three core domains—Technological Knowledge (TK), Pedagogical Knowledge (PK), and Content Knowledge (CK)—and their intersections, culminating in Technological Pedagogical Content Knowledge (TPACK) (Ifinedo et al., 2020; Mishra and Koehler, 2006; Syamdianita and Cahyono, 2021; Wu et al., 2015). In the literature, TPACK serves both as a theory of teacher competence and as a widely used framework for assessment. Instrument-development studies have repeatedly adapted and validated TPACK-based measures across different teacher populations and instructional settings (Anthony et al., 2021; Nurwahidah et al., 2023; Syamdianita and Cahyono, 2021). A consistent empirical pattern also appears in this literature: teachers often report stronger content and pedagogical knowledge than technology-related and technology-integration knowledge, highlighting integration itself as the central challenge (Hsu et al., 2023; Uygun et al., 2023).

A complementary perspective comes from DigCompEdu, which conceptualizes educator digital competence as a broader professional construct. This perspective includes professional communication and collaboration, digital resources, teaching and learning, and assessment (Sofwan et al., 2024). DigCompEdu-oriented findings show that pre-service teachers often perceive themselves as stronger in communication and resource management than in reflective practice and evidence use, indicating that digital competence includes professional and pedagogical dimensions as well as technical ones (Sofwan et al., 2024).

Self-efficacy provides a third theoretical foundation. Bandura (1977) defined self-efficacy as beliefs about one’s capability to organize and execute the actions required to attain particular outcomes and established its role in effort, persistence, behavioral choice, and responses to difficulty. Technology-related self-efficacy applies this capability-belief mechanism to technology-supported activity, and studies with teachers and pre-service teachers link stronger technology-integration self-efficacy with greater confidence, persistence, and willingness to use technology instructionally (Wang et al., 2004; Chang et al., 2024; Pradita et al., 2023; Sridana et al., 2024). Self-efficacy is therefore conceptually different from possessing Technological Knowledge or skill: it concerns the individual’s judgment that those capabilities can be mobilized successfully under relevant conditions. In the present framework, Teaching-Related Technology Self-Efficacy (TTSE) represents this belief-based component of technological competence for teaching and is not treated as direct evidence of successful teaching performance. Together, TPACK, DigCompEdu, and self-efficacy theory provide complementary knowledge-, professional-practice-, and capability-belief foundations for the six-dimensional construct examined in this study.

### 1.3 Conceptual dimensions of technological competence

Based on this literature, technological competence for teaching is best understood as a multidimensional construct.

- First, it includes Foundational Digital Tool Use. Pre-service teachers often begin with standard tools such as presentation software, office applications, learning platforms, laptops, and mobile devices. These tools form the operational base of technology-supported instruction (Abubakir and Alshaboul, 2023; Imania et al., 2022).
- Second, it includes Professional Digital Communication and Collaboration. Digital competence in teaching involves communication with students, peers, and professional communities through online platforms and collaborative environments. DigCompEdu-based evidence identifies this domain as a key component of educator competence (Sofwan et al., 2024).
- Third, it includes assessment, feedback, and evidence use. Research on pre-service online teaching frequently identifies assessment as a weak area, especially in relation to technology-supported feedback and the use of digital tools for monitoring learning (Abubakir and Alshaboul, 2023; Choi and Chung, 2025). This domain deserves separate attention because it directly affects instructional quality.
- Fourth, technological competence includes Pedagogically Aligned Technology Integration. TPACK research emphasizes that effective technology use depends on aligning tools with teaching methods and content goals rather than adding technology superficially (Ifinedo et al., 2020; Tan et al., 2023). This dimension reflects the core of technology-enhanced teaching.
- Fifth, it includes troubleshooting and adaptability. Technology-rich teaching requires teachers to respond to technical problems, contextual constraints, and changing classroom demands. Studies of instructional design and online teaching show that adaptation, reflection, and revision remain integral to successful technology use (Abubakir and Alshaboul, 2023; Wardani et al., 2020).
- Finally, technological competence includes Teaching-Related Technology Self-Efficacy. Confidence in using technology for instruction supports persistence, shapes classroom practice, and relates positively to TPACK and technology integration outcomes (Chang et al., 2024; Giles and Kent, 2016; Pradita et al., 2023; Sridana et al., 2024).

Taken together, these dimensions define technological competence for teaching as a multidimensional construct encompassing perceived operational capability, reported professional and assessment-related practices, Pedagogically Aligned Technology Integration, adaptive problem solving, and teaching-related self-efficacy. Their inclusion within one instrument does not imply that they are interchangeable manifestations of a single homogeneous ability or that a single higher-order factor is required. Accordingly, primary interpretation should focus on the six subscale scores.

**Figure 1:**
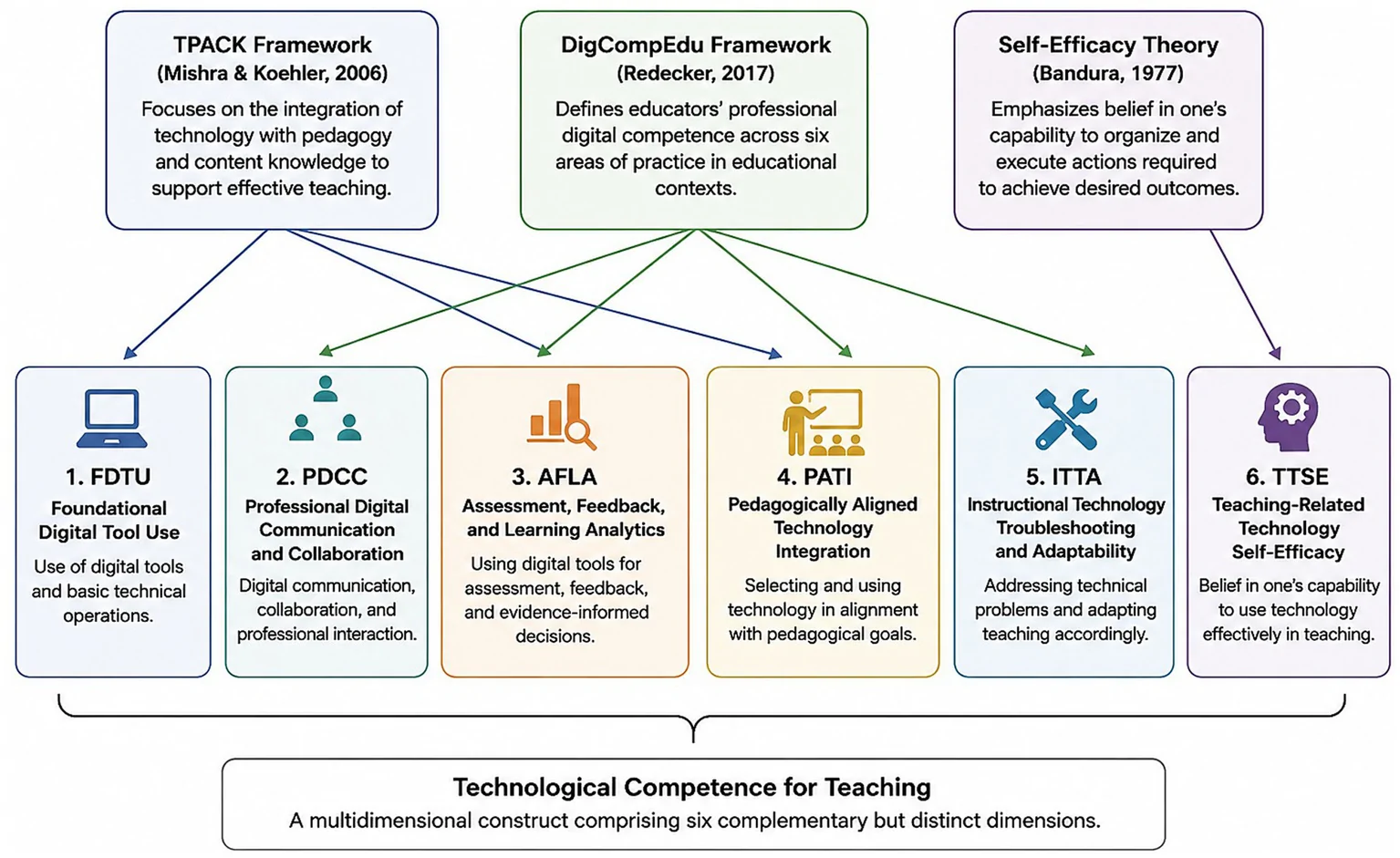
Theoretical foundations of the six dimensions of technological competence for teaching. TPACK, Technological Pedagogical Content Knowledge; DigCompEdu, European Framework for the Digital Competence of Educators; FDTU, Foundational Digital Tool Use; PDCC, Professional Digital Communication and Collaboration; AFLA, Assessment, Feedback, and Learning Analytics; PATI, Pedagogically Aligned Technology Integration; ITTA, Instructional Technology Troubleshooting and Adaptability; TTSE, Teaching-Related Technology Self-Efficacy. Arrows indicate the principal theoretical contribution of each framework to the proposed dimensions; they do not imply exclusive or causal relationships.

The proposed instrument integrates three complementary theoretical foundations. TPACK primarily informs technology–pedagogy alignment and technology-supported instructional decision making; DigCompEdu broadens the framework to include foundational digital practices, professional communication and collaboration, assessment and feedback, and adaptive use of digital technologies; and self-efficacy theory provides the capability-belief foundation represented by Teaching-Related Technology Self-Efficacy. As illustrated in Figure 1, these theoretical perspectives jointly inform six complementary but empirically distinguishable dimensions of technological competence for teaching.

### 1.4 Limitations of existing scales

Although many existing instruments assess technology integration through TPACK, several limitations remain. First, most scales focus strongly on knowledge domains and less on broader professional competence. TPACK-based instruments offer valuable measures of integrated knowledge, but they often do not capture dimensions such as digital communication, assessment practice, and troubleshooting in sufficient depth (Nurwahidah et al., 2023; Syamdianita and Cahyono, 2021).

Second, some validated instruments are highly context-specific. Scales designed for particular subject areas or instructional formats strengthen local validity, yet they also contribute to fragmentation in measurement and limit broader applicability (Anthony et al., 2021; Nurwahidah et al., 2023).

Third, DigCompEdu-oriented assessments address wider digital competence, but they do not provide the same subject-specific integration logic that TPACK offers. As a result, neither framework alone fully captures the range of competencies required for technology-enhanced teaching (Ifinedo et al., 2020; Sofwan et al., 2024).

Fourth, many instruments exclude self-efficacy, even though the literature consistently links it to technology adoption, persistence, and instructional behavior (Chang et al., 2024; Sridana et al., 2024). Omitting this dimension leaves an incomplete picture of technological competence.

Overall, the literature points to the need for a multidimensional instrument that integrates knowledge, practice, and belief-based components within a single framework.

Table 1 summarizes representative instruments aligned with the three theoretical traditions informing the present scale and illustrates both their contributions and the measurement gap addressed in this study.

**Table 1:** Comparison of representative measures related to TPACK, educator digital competence, and technology-integration self-efficacy.

Instrument	Source	Target population	Main dimensions	Items	Primary purpose
Survey of Preservice Teachers’ Knowledge of Teaching and Technology	Schmidt et al. (2009)	Pre-service teachers	TK, CK, PK, PCK, TCK, TPK, TPACK	47	Self-assessment of TPACK-related knowledge development
DigCompEdu Check-In	European DigCompEdu framework; validated by Redecker (2017) and Cabero-Almenara et al. (2020)	Educators / university teachers	Professional engagement, digital resources, teaching and learning, assessment, empowering learners, facilitating learners’ digital competence	22	Self-reflection on educators’ digital competence across six professional areas
Technology integration self-efficacy/computer technology integration survey	Wang et al. (2004)	Pre-service teachers	Technology-integration self-efficacy	21	Assess confidence in the ability to integrate technology into classroom instruction
Present scale	Current study	Pre-service teachers	FDTU, PDCC, AFLA, PATI, ITTA, TTSE	33	Integrate assessment of operational, professional, assessment-related, pedagogical, adaptive, and technology self-efficacy dimensions within a single subscale-profile measure

TK, Technological Knowledge; CK, Content Knowledge; PK, Pedagogical Knowledge; PCK, Pedagogical Content Knowledge; TCK, Technological Content Knowledge; TPK, Technological Pedagogical Knowledge; TPACK, Technological Pedagogical Content Knowledge; FDTU, Foundational Digital Tool Use; PDCC, Professional Digital Communication and Collaboration; AFLA, Assessment, Feedback, and Learning Analytics; PATI, Pedagogically Aligned Technology Integration; ITTA, Instructional Technology Troubleshooting and Adaptability; TTSE, Teaching-Related Technology Self-Efficacy.

### 1.5 Purpose of the study

In response to these gaps, this study aims to develop and validate a multidimensional self-report instrument for measuring pre-service teachers’ technological competence for teaching. This purpose follows directly from evidence showing uneven development across competence domains, recurring weaknesses in assessment-related technology use, and the importance of self-efficacy in technology integration (Abubakir and Alshaboul, 2023; Sofwan et al., 2024).

The study also follows established instrument-development practices in this field. Prior research commonly uses exploratory and confirmatory factor analysis to establish factorial structure and to test validity and reliability in technology integration scales (Anthony et al., 2021; Nurwahidah et al., 2023). A validated multidimensional instrument can provide differentiated information for research and teacher education by identifying comparatively stronger and weaker self-reported scores across technological competence domains. Such profiles may also support formative and program-level evaluation. However, longitudinal evidence is required before changes in subscale scores can be interpreted as responses to preparation or training.

## 2 Method

### 2.1 Research design

This study used a multi-phase scale development and validation design to assess pre-service teachers’ self-reported technological competence. The instrument was grounded in an integration of TPACK, DigCompEdu, and technology-related self-efficacy. The validation process followed standard procedures, including item development, expert review, exploratory factor analysis (EFA), confirmatory factor analysis (CFA), and validity and reliability testing (Anthony et al., 2021; Nurwahidah et al., 2023). Two independent samples were used to ensure cross-validation.

### 2.2 Participants

Participants were pre-service teachers enrolled in teacher-education programs at a public university in Türkiye. Participation was voluntary, and students were informed that their responses would be used solely for research purposes and analyzed in aggregate form. Participants were recruited from the eligible student population through announcements distributed within the teacher-education programs.

Two independently recruited samples were used. The pilot sample comprised 513 pre-service teachers and was used for item reduction and exploratory factor analysis. The main sample comprised 974 pre-service teachers and was reserved for confirmatory factor analysis, reliability and construct-validity analyses, gender measurement-invariance testing, and examination of concurrent associations with the scenario-based judgment measure.

The pilot and main samples were drawn from two different cohorts and therefore consisted of different individuals. Data for both cohorts were collected during April 2026. Because both cohorts were recruited from the same university and teacher-education setting, independence in the present study refers to non-overlapping participant membership. The present analyses did not evaluate differences between the cohorts in instructional exposure, coursework, instructors, or other cohort-specific institutional conditions.

Prior to analysis, responses were screened for completeness and missing data. Only cases with sufficient usable data were retained for analysis, resulting in final analytic samples of 513 participants for the pilot/EFA stage and 974 participants for the main/CFA stage. The exact number of initially submitted responses excluded during screening was not retained in the available study records and therefore cannot be reported reliably.

### 2.3 Instrument development

Item development followed a deductive, theory-driven approach in which the construct domains were specified before item writing. Deductive item generation is appropriate when a target construct is grounded in an established theoretical literature and items are developed to represent predefined conceptual domains (Hinkin, 1998). The construct map for the present instrument integrated three complementary perspectives: TPACK, DigCompEdu, and technology-related self-efficacy. TPACK informed pedagogically aligned technology use and the integration of technology with teaching and content knowledge (Ifinedo et al., 2020; Schmidt et al., 2009; Wu et al., 2015). DigCompEdu informed foundational digital activity, professional communication and collaboration, technology-supported assessment and feedback, and broader educator digital practices (Cabero-Almenara et al., 2020; Sofwan et al., 2024). Bandura’s self-efficacy theory and subsequent technology-integration self-efficacy research informed the capability-belief dimension represented by Teaching-Related Technology Self-Efficacy (TTSE) (Bandura, 1977; Wang et al., 2004).

On the basis of this theoretical construct map, the authors generated an intentionally broad initial pool of 90 candidate items distributed across the six predefined dimensions. The item-generation process was deductive rather than inductive: the dimensions and candidate items were derived from the theoretical frameworks and relevant literature rather than from interviews, focus groups, essays, open-ended responses, or other qualitative procedures. The initial pool was deliberately broader than the intended final instrument so that the conceptual breadth of each dimension could be represented before expert review and subsequent psychometric reduction, consistent with established recommendations for scale development (Hinkin, 1998; Worthington and Whittaker, 2006).

The 90 candidate items were prepared in parallel English and Turkish versions. Both versions were reviewed for conceptual clarity, linguistic appropriateness, correspondence of meaning, and suitability for pre-service teachers. During the subsequent content-review process, reviewers also evaluated the relevance of each item to its intended dimension, as well as its wording, clarity, and appropriateness for the target population. Accordingly, the cross-language evidence in the present study consists of parallel item preparation and qualitative review of linguistic appropriateness and correspondence of meaning. The study did not evaluate quantitative cross-language measurement equivalence. Turkish was the language of instruction at the participating university and the language in which the instrument was administered. Responses were recorded on a five-point Likert-type scale.

Content validity was subsequently evaluated by two internal reviewers—the authors who had participated in construct definition and item development—and seven external experts with relevant disciplinary expertise. Each reviewer independently evaluated the relevance of the candidate items to their intended dimensions and considered wording, clarity, and suitability for the target population. Because the two authors had been directly involved in construct definition and item generation, they were not treated as independent external experts. Accordingly, the reported content-validity indices represent evidence derived from combined internal and external review rather than ratings from nine independent external experts. The complete bilingual 90-item pool and item-retention decisions are provided in Appendix A, and the item-level content-validity ratings and retention decisions are provided in Appendix B.

### 2.4 Scoring

Items are rated on a five-point Likert-type scale ranging from 1 (strongly disagree) to 5 (strongly agree). Subscale scores are calculated as the mean of the retained items assigned to each dimension: FDTU (FDTU01–FDTU06), PDCC (PDCC01, PDCC02, PDCC03, PDCC05, PDCC06, and PDCC08), AFLA (AFLA03–AFLA08), PATI (PATI01, PATI03, PATI04, PATI06, and PATI07), ITTA (ITTA02, ITTA03, ITTA06, ITTA07, and ITTA08), and TTSE (TTSE01, TTSE02, TTSE03, TTSE04, and TTSE06). Higher subscale scores indicate stronger self-reported technological competence within the corresponding dimension. Primary interpretation should be based on the six subscale scores rather than an overall score because the instrument is multidimensional and several inter-factor associations are weak or negligible. Because only complete usable responses were included in the present analyses, no missing-item scoring procedure was required.

### 2.5 Exploratory factor analysis

EFA was conducted using the pilot sample. Data suitability was evaluated using the Kaiser–Meyer–Olkin measure and Bartlett’s test of sphericity. Principal axis factoring was used because the objective was to identify common latent dimensions and the retained indicators showed departure from multivariate normality. Promax, an oblique rotation, was selected because the six dimensions were theoretically permitted to correlate; oblique rotation allows the empirical solution to estimate those relations rather than imposing zero factor correlations. Importantly, selecting an oblique rotation does not require the resulting correlations to be large. Factor-retention decisions were based jointly on parallel analysis, scree-plot inspection, and theoretical interpretability rather than on a single retention rule (Fabrigar et al., 1999; Costello and Osborne, 2005; Worthington and Whittaker, 2006).

Item reduction was conducted iteratively using prespecified statistical criteria together with conceptual interpretability. Items were considered for removal when uniqueness exceeded 0.60, the primary factor loading was below 0.55 or above 0.85, or the difference between the absolute values of the two largest pattern coefficients was below 0.20. These criteria were used to promote adequate common-factor representation, clear primary loadings, and empirical separation from competing factors. In the present analysis, all 15 deleted items were removed because their uniqueness values exceeded 0.60; none required removal because of a primary loading below 0.55, a primary loading above 0.85, or insufficient separation between the two largest absolute pattern coefficients. The resulting final solution contained 33 items across six factors and explained 62.8% of total variance.

### 2.6 Confirmatory factor analysis

CFA was conducted with the independent main sample to evaluate the six-factor structure identified through EFA. The model specified six correlated latent variables, with retained items loading on their intended factors. Because the indicators were ordinal, the model was estimated using diagonally weighted least squares (DWLS). Model fit was evaluated using *χ*^2^, CFI, TLI, RMSEA, and SRMR, with primary emphasis on approximate fit indices.

Modification indices were not examined. No correlated residuals, cross-loadings, item deletions, or other *post-hoc* model modifications were introduced after estimation. The reported model therefore represents the prespecified six-factor structure derived from the exploratory analysis.

### 2.7 Validity and reliability

Convergent validity was evaluated using average variance extracted (AVE), and discriminant validity was examined using heterotrait–monotrait ratios (HTMT). Internal consistency was assessed using McDonald’s omega and Cronbach’s alpha. Concurrent associations with scenario-based technological-pedagogical judgment were examined using Pearson correlations and multiple linear regression. Because the self-report scale and scenario-based quiz were administered concurrently and were conceptually aligned, these analyses were not interpreted as evidence of prospective predictive validity. Gender measurement invariance was evaluated using multigroup CFA for ordered categorical indicators.

### 2.8 Concurrent associations with scenario-based judgment

Concurrent associations were examined using an 18-item scenario-based quiz assessing technological-pedagogical judgment across the six competence domains, with three items per domain. Each item presented a teaching-related scenario with five response options and one most appropriate answer. Correct responses were scored 1 and incorrect responses 0, yielding total scores from 0 to 18. The complete bilingual quiz and answer key are provided in Appendix C.

Because the quiz was developed within the same research program, reflected the same six conceptual domains, and was administered concurrently with the self-report scale, it was treated as a framework-aligned judgment measure rather than an independent external criterion or prospective predictive measure. The quiz demonstrated satisfactory internal consistency in the main sample (McDonald’s *ω* = 0.870; Cronbach’s *α* = 0.864).

Pearson correlations and multiple linear regression were used to examine the concurrent associations of the six technological competence dimensions with the total quiz score. In response to the domain structure of the quiz, exploratory domain-matched correlations were also examined between each self-report subscale and the corresponding three-item scenario-domain score. Because each domain score was based on only three dichotomously scored items, these analyses were treated as exploratory and the domain scores were not interpreted as established psychometric subscores. Subscore research shows that useful standalone subscores require adequate measurement information and sufficient distinctiveness from the overall score (Haberman, 2008; Sinharay et al., 2011). Accordingly, the 18-item total quiz score remained the primary concurrent criterion.

### 2.9 Measurement invariance

Measurement invariance across gender was examined using multigroup CFA for ordered categorical indicators with DWLS estimation. Configural invariance evaluated whether the same six-factor structure was represented across groups. Loading invariance constrained corresponding factor loadings to equality, and threshold invariance additionally constrained corresponding item thresholds to equality. Invariance decisions were evaluated primarily from changes in approximate fit indices across successive models.

### 2.10 Descriptive analyses and cross-sample comparisons

Descriptive analyses were conducted to examine construct distributions and cross-sample stability. Construct means were calculated using retained items, and Welch’s *t* tests were used to compare samples. Effect sizes were computed to assess practical significance. Gender-stratified analyses were conducted following threshold invariance, and item-level statistics were examined to evaluate response consistency across samples.

### 2.11 Statistical software

All analyses were conducted using JASP version 0.96.0.0. Exploratory factor analysis, descriptive statistics, reliability analyses, group comparisons, correlations, and regression analyses were conducted in JASP using procedures appropriate for psychometric scale validation. Confirmatory factor analysis and measurement invariance testing were also conducted in JASP using structural equation modeling procedures with DWLS estimation for ordinal indicators. The same retained 33-item structure was used across CFA, reliability, validity, and invariance analyses to ensure consistency across analytic stages.

### 2.12 Ethical considerations

Participation was voluntary, and participants were informed about the purpose of the study before completing the instrument. Responses were used only for research purposes and were analyzed in aggregate form. No identifying information was reported in the manuscript. The study followed ethical principles for research with human participants.

## 3 Results

### 3.1 Sample comparability between pilot and main samples

The pilot (EFA; *n* = 513) and main (CFA; *n* = 974) samples were comparable across all examined variables (Table 2). There was no significant difference in age, *t*(1485) = 0.32, *p* = 0.749, *d* = 0.02. Similarly, distributions of gender, *χ*^2^(1) = 0.27, *p* = 0.602, *V* = 0.01; year of study, *χ*^2^(6) = 3.28, *p* = 0.773, *V* = 0.05; number of siblings, *χ*^2^(5) = 3.35, *p* = 0.645, *V* = 0.05; and marital status, *χ*^2^(1) = 0.18, *p* = 0.675, *V* = 0.01, did not differ significantly.

**Table 2:** Demographic profiles and statistical comparability of the pilot and main study samples.

Variable	Pilot (EFA; *n* = 513)	Main (CFA; *n* = 974)	Sample comparability
Age (years), M (SD)	24.45 (6.11)	24.35 (6.04)	*t* = 0.32, *p* = 0.749, *d* = 0.02
Gender, *n* (%)			*χ*^2^(1) = 0.27, *p* = 0.602, *V* = 0.01
Male	190 (37.0)	346 (35.5)	
Female	323 (63.0)	628 (64.5)	
Year of study, *n* (%)			*χ*^2^(6) = 3.28, *p* = 0.773, *V* = 0.05
1st year	56 (10.9)	118 (12.1)	
2nd year	277 (54.0)	518 (53.2)	
3rd year	80 (15.6)	172 (17.7)	
4th year	94 (18.3)	157 (16.1)	
Preparatory	1 (0.2)	2 (0.2)	
Thesis Master’s	1 (0.2)	3 (0.3)	
Non-Thesis Master’s	4 (0.8)	4 (0.4)	
Number of siblings, *n* (%)			*χ*^2^(5) = 3.35, *p* = 0.645, *V* = 0.05
0	11 (2.1)	14 (1.4)	
1	17 (3.3)	49 (5.0)	
2	53 (10.3)	102 (10.5)	
3	76 (14.8)	147 (15.1)	
4	75 (14.6)	139 (14.3)	
5 or more	281 (54.8)	523 (53.7)	
Marital status, *n* (%)			*χ*^2^(1) = 0.18, *p* = 0.675, *V* = 0.01
Single	450 (87.7)	863 (88.6)	
Married	63 (12.3)	111 (11.4)	

Effect sizes were negligible, indicating substantial demographic comparability between the samples on the variables examined. These comparisons address demographic similarity but do not establish equivalence in unmeasured cohort-specific instructional or institutional experiences.

### 3.2 Content validity assessment

Content validity was evaluated using ratings from two internal reviewers, who were the authors involved in item development, and seven external experts. Across the initial 90-item pool, I-CVI values ranged from 0.11 to 1.00. All 48 items selected for pilot administration met the prespecified item-level content-validity criterion. At the construct level, S-CVI/Ave values based on the original item pool were 0.63 for FDTU, 0.67 for PDCC, 0.59 for AFLA, 0.61 for PATI, 0.64 for ITTA, and 0.61 for TTSE. When only the 48 items selected for pilot administration were considered, S-CVI/Ave values increased to 0.88, 0.90, 0.89, 0.88, 0.93, and 0.90, respectively. Because the two internal reviewers had participated in item development, these indices represent content-validity evidence derived from combined internal and external review rather than ratings from nine independent external experts.

Following iterative EFA item reduction, 33 items were retained: FDTU (FDTU01–FDTU06; 6 items), PDCC (PDCC01, PDCC02, PDCC03, PDCC05, PDCC06, and PDCC08; 6 items), AFLA (AFLA03–AFLA08; 6 items), PATI (PATI01, PATI03, PATI04, PATI06, and PATI07; 5 items), ITTA (ITTA02, ITTA03, ITTA06, ITTA07, and ITTA08; 5 items), and TTSE (TTSE01, TTSE02, TTSE03, TTSE04, and TTSE06; 5 items).

### 3.3 Exploratory factor analysis

An exploratory factor analysis (EFA) was initiated with the 48 candidate items retained following content review. Items were evaluated iteratively according to the prespecified statistical and conceptual criteria, resulting in a final 33-item solution. Unless otherwise indicated, the factorability diagnostics, factor-retention results, and final factor statistics reported below refer to the retained 33-item solution.

#### 3.3.1 Preliminary analyses

Preliminary diagnostics for the final 33-item solution indicated that the retained item set was suitable for factor analysis (Table 3). The Kaiser–Meyer–Olkin (KMO) measure of sampling adequacy was 0.851, with item-level KMO values ranging from 0.783 to 0.896. Bartlett’s test of sphericity was statistically significant, *χ*^2^(528) = 10385.60, *p* < 0.001, indicating that the correlation matrix was factorable.

**Table 3:** Factorability diagnostics for the exploratory factor analysis.

Statistic	Value
Kaiser–Meyer–Olkin (KMO)	0.851
Item-level KMO range	0.783–0.896
Bartlett’s test of sphericity	*χ*^2^(528) = 10385.60, *p* < 0.001
Mardia’s multivariate skewness	*χ*^2^(6545) = 7336.76, *p* < 0.001
Small-sample skewness correction	*χ*^2^(6545) = 7382.20, *p* < 0.001
Mardia’s multivariate kurtosis	*p* = 0.140
Model chi-square (final solution)	*χ*^2^(345) = 827.75, *p* < 0.001

KMO = Kaiser–Meyer–Olkin measure of sampling adequacy.

Multivariate normality was evaluated for the retained 33 items using Mardia’s tests. The multivariate skewness statistic was significant, *χ*^2^(6545) = 7336.76, *p* < 0.001, and the corresponding small-sample corrected statistic was also significant, *χ*^2^(6545) = 7382.20, *p* < 0.001, indicating departure from multivariate normality in terms of skewness. In contrast, multivariate kurtosis was not statistically significant (*p* = 0.140). Given this evidence of nonnormality, principal axis factoring was retained as the extraction method.

For the final six-factor, 33-item solution, the model chi-square was statistically significant, *χ*^2^(345) = 827.75, *p* < 0.001. Because chi-square is sensitive to sample size and model complexity, this statistic was interpreted together with the factor-retention evidence, loading pattern, uniqueness values, and theoretical interpretability rather than used as a stand-alone criterion for model adequacy.

#### 3.3.2 Factor retention

**Figure 2:**
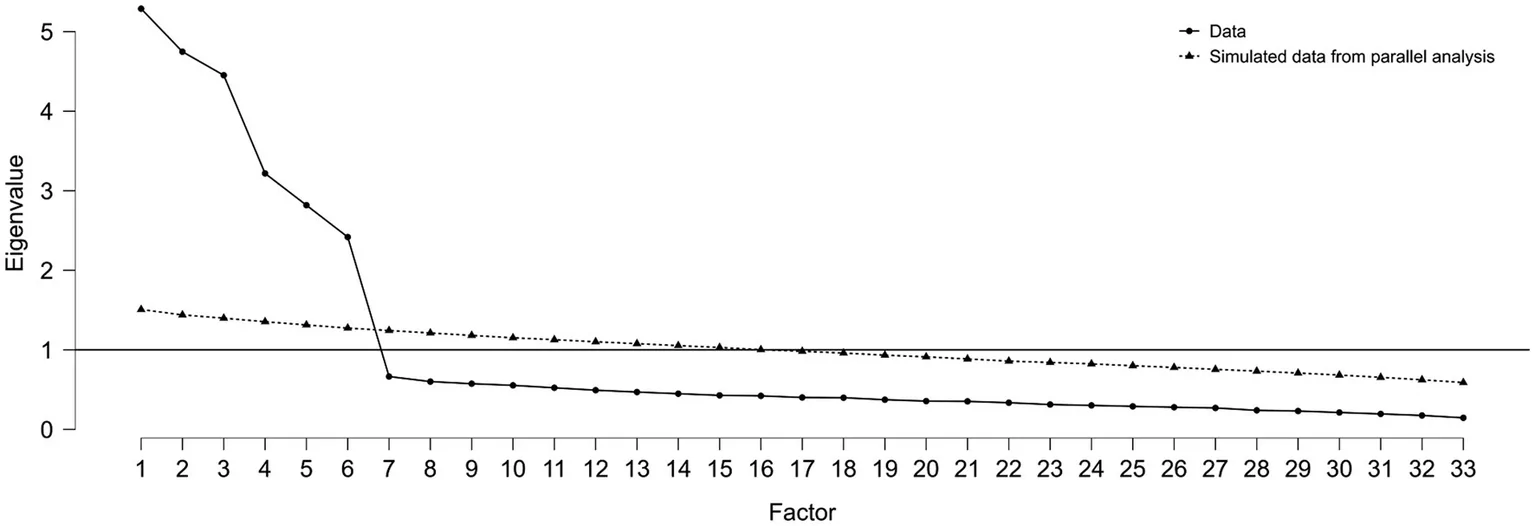
Parallel analysis scree plot. The scree plot shows a clear inflection after the sixth factor, supporting the retention of six factors.

Factor retention was determined using parallel analysis, scree plot inspection, and theoretical interpretability. As shown in Table 4, the first six empirical eigenvalues exceeded the corresponding simulated eigenvalues, whereas the seventh did not. The scree plot (Figure 2) also showed a clear break after the sixth factor, supporting retention of a six-factor solution.

**Table 4:** Parallel analysis results for factor retention.

Factor	Real data eigenvalue	Simulated eigenvalue
1	5.288	1.517
2	4.748	1.450
3	4.452	1.395
4	3.218	1.353
5	2.819	1.317
6	2.418	1.282
7	0.665	1.243

Factors were retained when empirical eigenvalues exceeded simulated eigenvalues.

#### 3.3.3 Factor characteristics

The unrotated eigenvalues for the retained factors were 4.918, 4.400, 4.069, 2.864, 2.439, and 2.035, explaining 14.9, 13.3, 12.3, 8.7, 7.4, and 6.2% of total variance, respectively (Table 5). Together, these factors accounted for 62.8% of total variance. After promax rotation, variance was more evenly distributed across factors (11.8, 11.1, 11.1, 10.3, 10.0, and 8.5%).

**Table 5:** Factor characteristics for the final six-factor solution.

Factor	Unrotated solution			Rotated solution		
	Eigenvalue	Proportion	Cumulative	Sum Sq. loadings	Proportion	Cumulative
1	4.918	0.149	0.149	3.889	0.118	0.118
2	4.400	0.133	0.282	3.677	0.111	0.229
3	4.069	0.123	0.406	3.649	0.111	0.340
4	2.864	0.087	0.492	3.400	0.103	0.443
5	2.439	0.074	0.566	3.296	0.100	0.543
6	2.035	0.062	0.628	2.813	0.085	0.628

#### 3.3.4 Item reduction

The 48 candidate items were evaluated iteratively using prespecified statistical criteria together with conceptual interpretability. Items were considered for removal when uniqueness exceeded 0.60, the primary factor loading was below 0.55 or above 0.85, or the difference between the absolute values of the two largest pattern coefficients was below 0.20. Fifteen items were removed during this process. All 15 deletions resulted from uniqueness values exceeding 0.60, indicating that more than 60% of the variance in those items was not accounted for by the common-factor solution. None of the deleted items required removal because of a primary loading below 0.55, a primary loading above 0.85, or insufficient separation between the two largest absolute pattern coefficients.

The remaining 33 items satisfied the final statistical criteria and provided conceptually interpretable representation of the six intended dimensions. Item retention was not uniform across dimensions: FDTU, PDCC, and AFLA retained six items each, whereas PATI, ITTA, and TTSE retained five items each. Accordingly, although all six intended dimensions remained represented in the final solution, the reduction process resulted in narrower item representation for some subscales than for others. The present analyses do not establish whether every facet represented in the larger item pool remained equally represented in the final instrument. Subsequent content-validation studies should examine the breadth of content represented by each retained subscale.

#### 3.3.5 Final factor structure

The final EFA solution yielded six factors, corresponding to:

1. Foundational Digital Tool Use (FDTU)
2. Professional Digital Communication and Collaboration (PDCC)
3. Assessment, Feedback, and Learning Analytics (AFLA)
4. Pedagogically Aligned Technology Integration (PATI)
5. Instructional Technology Troubleshooting and Adaptability (ITTA)
6. Teaching-Related Technology Self-Efficacy (TTSE)

All retained items showed strong primary loadings ranging from 0.675 to 0.847, with no problematic cross-loadings. The complete pattern matrix for the final 33-item exploratory factor solution is provided in Appendix D.

#### 3.3.6 Factor correlations

Promax rotation produced factor correlations ranging from −0.054 to 0.333 (Table 6). Several associations were weak or negligible, whereas a smaller number were modestly positive. The observed pattern does not conflict with the use of oblique rotation: Promax allowed the factors to correlate where supported by the data without imposing orthogonality, and the resulting estimates showed that only selected dimensions were meaningfully related. This outcome is consistent with the theoretical specification of six complementary but noninterchangeable domains. The weak and near-zero associations therefore provide empirical evidence of differentiation among several dimensions and support primary interpretation at the subscale level rather than as manifestations of a single homogeneous general factor (Fabrigar et al., 1999).

**Table 6:** Factor correlations (promax rotation).

Factor	1	2	3	4	5	6
1	1.000					
2	−0.004	1.000				
3	−0.028	0.069	1.000			
4	−0.029	−0.022	0.333	1.000		
5	0.202	−0.019	0.009	0.082	1.000	
6	0.074	0.253	0.094	0.108	−0.054	1.000

### 3.4 Confirmatory factor analysis

A confirmatory factor analysis (CFA) was conducted to test the hypothesized six-factor measurement model. The hypothesis was derived jointly from the *a priori* theoretical framework and the six-factor structure retained in the independent EFA sample. The model specified six correlated latent variables representing FDTU, PDCC, AFLA, PATI, ITTA, and TTSE, with their respective indicators loading on their intended factors.

#### 3.4.1 Model fit

The CFA was estimated using diagonally weighted least squares (DWLS) because the retained indicators were ordinal. The six-factor model produced fit indices consistent with the proposed structure, *χ*^2^(480) = 807.32, *p* < 0.001, CFI = 0.980, TLI = 0.978, RMSEA = 0.026, 90% CI [0.023, 0.030], and SRMR = 0.036. Additional indices were GFI = 0.992, MFI = 0.912, RNI = 0.980, and NFI = 0.953. Taken together, these indices provided support for the adequacy of the proposed six-factor representation (Table 7).

**Table 7:** Model fit indices for the six-factor confirmatory factor analysis.

Index	Value	Index	Value
*χ* ^2^	807.32	RNI	0.980
df	480	RMSEA	0.026
*p*	<0.001	RMSEA 90% CI	[0.023, 0.030]
CFI	0.980	SRMR	0.036
TLI	0.978	GFI	0.992
NFI	0.953	MFI	0.912

Estimator = DWLS. Fit indices are scaled due to categorical indicators.

**Figure 3:**
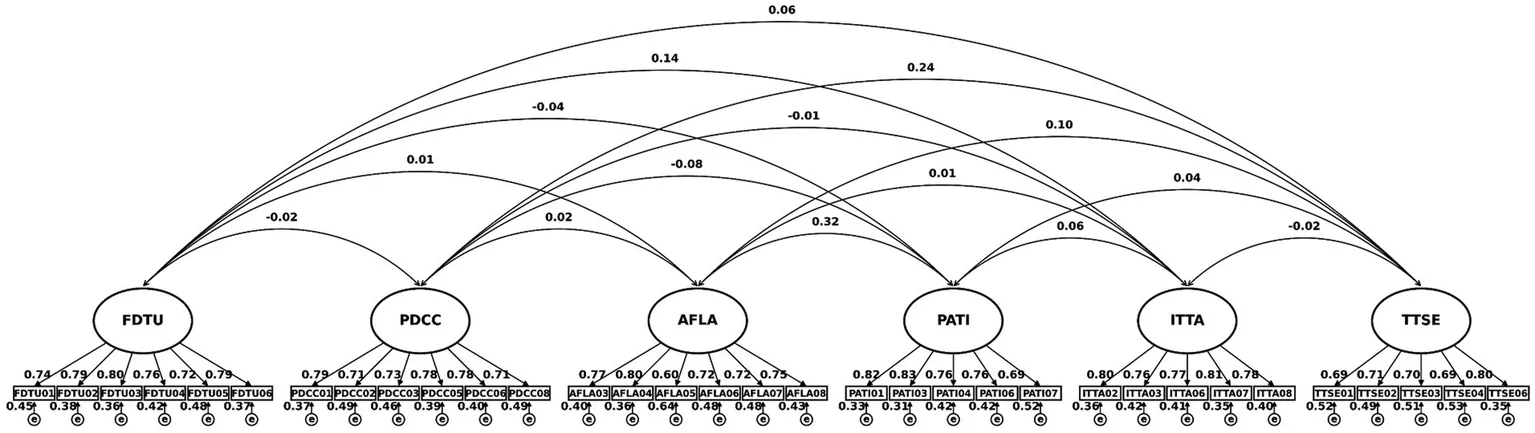
Model plot for the six-factor measurement model.

The standardized solution is presented in Figure 3 to illustrate the magnitude of factor loadings and the pattern of correlations among the six latent constructs (FDTU, PDCC, AFLA, PATI, ITTA, TTSE) and their associated indicators. Standardized factor loadings are displayed on the paths from latent variables to observed indicators.

#### 3.4.2 Factor loadings

All items loaded significantly on their intended latent factors (all *p* < 0.001). The unstandardized estimates ranged from 0.777 to 1.161, whereas the squared standardized loadings ranged from 0.362 to 0.695. Because the final column of Table 8 reports squared standardized loadings (*λ*^2^), these values were used to compute the AVE estimates reported in Table 9. The corresponding standardized loadings (*λ*), obtained by taking the square roots of these values, ranged approximately from 0.602 to 0.834, indicating moderate to strong associations between the observed indicators and their respective latent constructs. Overall, the results suggest that the retained items provide acceptable indicators of the six latent dimensions, while the pattern of estimates supports the distinctiveness of the constructs. Table 8 presents the unstandardized estimates and squared standardized loadings from the confirmatory factor analysis, with one loading per factor fixed to 1.000 for model identification.

**Table 8:** Unstandardized CFA factor loadings and squared standardized loadings.

Factor	Indicator	Unstd. Estimate	SE	*z*	*p*	95% CI (Unstd.)	Squared Std. loading (*λ*^2^)
FDTU	FDTU01	1.000	0.000	—	—	[1.000, 1.000]	0.553
	FDTU02	1.061	0.033	31.80	<0.001	[0.995, 1.126]	0.622
	FDTU03	1.077	0.032	33.26	<0.001	[1.013, 1.140]	0.641
	FDTU04	1.022	0.036	28.52	<0.001	[0.952, 1.092]	0.577
	FDTU05	0.969	0.037	26.12	<0.001	[0.896, 1.042]	0.519
	FDTU06	1.064	0.033	32.54	<0.001	[1.000, 1.128]	0.626
PDCC	PDCC01	1.000	0.000	—	—	[1.000, 1.000]	0.626
	PDCC02	0.903	0.034	26.77	<0.001	[0.837, 0.969]	0.510
	PDCC03	0.926	0.033	28.13	<0.001	[0.862, 0.991]	0.537
	PDCC05	0.987	0.031	32.27	<0.001	[0.927, 1.047]	0.609
	PDCC06	0.981	0.030	32.95	<0.001	[0.923, 1.039]	0.602
	PDCC08	0.900	0.034	26.14	<0.001	[0.832, 0.967]	0.506
AFLA	AFLA03	1.000	0.000	—	—	[1.000, 1.000]	0.599
	AFLA04	1.035	0.028	37.39	<0.001	[0.981, 1.089]	0.642
	AFLA05	0.777	0.036	21.56	<0.001	[0.707, 0.848]	0.362
	AFLA06	0.935	0.034	27.82	<0.001	[0.869, 1.001]	0.524
	AFLA07	0.930	0.034	27.04	<0.001	[0.862, 0.997]	0.518
	AFLA08	0.973	0.032	30.59	<0.001	[0.911, 1.036]	0.568
PATI	PATI01	1.000	0.000	—	—	[1.000, 1.000]	0.671
	PATI03	1.017	0.022	45.26	<0.001	[0.973, 1.061]	0.695
	PATI04	0.932	0.027	34.82	<0.001	[0.879, 0.984]	0.583
	PATI06	0.927	0.027	33.90	<0.001	[0.874, 0.981]	0.578
	PATI07	0.847	0.029	29.57	<0.001	[0.791, 0.903]	0.482
ITTA	ITTA02	1.000	0.000	—	—	[1.000, 1.000]	0.636
	ITTA03	0.957	0.032	30.08	<0.001	[0.895, 1.020]	0.583
	ITTA06	0.968	0.032	30.71	<0.001	[0.906, 1.029]	0.595
	ITTA07	1.010	0.026	39.02	<0.001	[0.959, 1.061]	0.649
	ITTA08	0.974	0.026	37.71	<0.001	[0.923, 1.024]	0.603
TTSE	TTSE01	1.000	0.000	—	—	[1.000, 1.000]	0.478
	TTSE02	1.033	0.050	20.76	<0.001	[0.936, 1.131]	0.511
	TTSE03	1.014	0.052	19.34	<0.001	[0.911, 1.117]	0.492
	TTSE04	0.997	0.051	19.46	<0.001	[0.896, 1.097]	0.475
	TTSE06	1.161	0.052	22.14	<0.001	[1.058, 1.263]	0.645

Unstandardized loadings are reported using marker-variable identification (first loading fixed to 1.000 within each factor). All factor loadings were statistically significant (*p* < 0.001).

**Table 9:** Average variance extracted (AVE).

Factor	AVE
FDTU	0.590
PDCC	0.565
AFLA	0.536
PATI	0.602
ITTA	0.613
TTSE	0.520

#### 3.4.3 Factor variances and covariances

All latent factor variance estimates were statistically significant (ps < 0.001), indicating that each latent construct showed sufficient variability in the CFA model. These estimates are reported to document model estimation and latent construct variability; they are not presented as the primary evidence for construct validity (Table 10).

**Table 10:** Latent factor variance estimates from the CFA model.

Factor	Estimate	SE	*z*	*p*	95% CI lower	95% CI upper
FDTU	0.553	0.029	18.76	<0.001	0.495	0.610
PDCC	0.626	0.027	22.87	<0.001	0.572	0.679
AFLA	0.599	0.025	23.86	<0.001	0.550	0.649
PATI	0.671	0.024	28.45	<0.001	0.625	0.718
ITTA	0.636	0.025	25.52	<0.001	0.587	0.685
TTSE	0.478	0.036	13.37	<0.001	0.408	0.549

Estimates are latent factor variance estimates from the CFA model under the model’s identification and scaling approach. These values document statistically significant variability in the latent constructs. They should not be interpreted as AVE values or as item-level squared standardized loadings.

The substantive interpretation of the measurement model is based mainly on model fit, item-level squared standardized loadings, AVE, HTMT, factor covariances, reliability, and measurement invariance results, which will be presented sequentially.

Factor covariances were examined to characterize relationships among the six dimensions. The results showed a heterogeneous pattern of associations: several factor pairs were significantly related, whereas other relationships were weak or nonsignificant. The largest positive associations involved AFLA with PATI and PDCC with TTSE (Table 11). This pattern is consistent with the interpretation of technological competence for teaching as a multidimensional profile of distinguishable subscales.

**Table 11:** Factor covariances.

Factor pair	Estimate	SE	*z*	*p*
FDTU ↔ ITTA	0.085	0.021	3.969	<0.001
PDCC ↔ TTSE	0.133	0.021	6.227	<0.001
AFLA ↔ PATI	0.201	0.022	8.936	<0.001
AFLA ↔ TTSE	0.054	0.020	2.694	0.007
PDCC ↔ PATI	−0.051	0.025	−2.098	0.036

Only statistically significant covariances are displayed.

#### 3.4.4 Convergent validity

Convergent validity was evaluated using average variance extracted (AVE). AVE values ranged from 0.520 to 0.613, exceeding the recommended 0.50 threshold (Table 9). These results indicate that each latent construct explained more than half of the variance in its indicators.

#### 3.4.5 Discriminant validity

Discriminant validity was evaluated using the heterotrait–monotrait ratio (HTMT). HTMT values ranged from 0.024 to 0.310 and remained below the conventional 0.85 threshold, indicating that the six dimensions were empirically distinguishable (Table 12). The particularly low HTMT values observed for several factor pairs should be interpreted together with the weak inter-factor correlations and the multidimensional, subscale-focused representation of technological competence for teaching.

**Table 12:** HTMT ratios.

Factor	FDTU	PDCC	AFLA	PATI	ITTA	TTSE
FDTU	—					
PDCC	0.039	—				
AFLA	0.028	0.046	—			
PATI	0.042	0.074	0.310	—		
ITTA	0.115	0.024	0.035	0.066	—	
TTSE	0.059	0.221	0.082	0.034	0.035	—

#### 3.4.6 Explained variance

Item-level *R*^2^ values ranged from 0.362 to 0.695, indicating moderate to substantial explained variance across indicators.

### 3.5 Measurement invariance

Measurement invariance across gender was examined using multi-group CFA with DWLS estimation. The configural model showed fit indices consistent with the proposed structure, *χ*^2^(960) = 1264.25, *p* < 0.001, CFI = 0.981, TLI = 0.979, RMSEA = 0.026, 90% CI [0.022, 0.029], and SRMR = 0.045, supporting the equivalence of the six-factor structure across female and male groups. The loading-invariance model also showed comparable fit, *χ*^2^(987) = 1288.46, *p* < 0.001, CFI = 0.981, TLI = 0.980, RMSEA = 0.025, 90% CI [0.021, 0.029], and SRMR = 0.046. Relative to the configural model, changes in fit were negligible (ΔCFI = 0.000, ΔRMSEA = −0.001, ΔSRMR = 0.001), supporting metric invariance. The threshold-invariance model likewise produced comparable fit, *χ*^2^(981) = 1281.54, *p* < 0.001, CFI = 0.981, TLI = 0.980, RMSEA = 0.025, 90% CI [0.021, 0.029], and SRMR = 0.046. Compared with the metric model, changes in fit were zero or trivial (ΔCFI = 0.000, ΔRMSEA = 0.000, ΔSRMR = 0.000), supporting threshold invariance across gender. Overall, the findings indicate that configural, loading, and threshold invariance were supported across gender, suggesting that structural relations and latent mean comparisons are appropriate (Table 13).

**Table 13:** Measurement invariance across gender.

Model	*χ* ^2^	df	CFI	TLI	RMSEA	90% CI	SRMR	ΔCFI	ΔRMSEA	ΔSRMR	Decision
Configural	1264.25	960	0.981	0.979	0.026	[0.022, 0.029]	0.045	—	—	—	Supported
Loading invariance (metric)	1288.46	987	0.981	0.980	0.025	[0.021, 0.029]	0.046	0.000	−0.001	0.001	Supported
Threshold invariance (scalar)	1281.54	981	0.981	0.980	0.025	[0.021, 0.029]	0.046	0.000	0.000	0.000	Supported

Models were estimated using DWLS for ordered categorical indicators. ΔCFI, ΔRMSEA, and ΔSRMR compare each model with the immediately preceding model. The reduction from df = 987 in the loading-invariance model to df = 981 in the threshold-invariance model was verified against the original software output. Under threshold invariance, six latent-factor means in the nonreference group became freely estimated while the corresponding reference-group means remained fixed to zero for identification. Releasing these six parameters accounts for the six-degree-of-freedom decrease. Invariance decisions were based primarily on changes in approximate fit indices.

### 3.6 Concurrent associations with scenario-based judgment

The scenario-based quiz demonstrated satisfactory internal consistency in the main sample (McDonald’s *ω* = 0.870; Cronbach’s *α* = 0.864). Concurrent associations between quiz scores and the six technological competence dimensions were examined using Pearson correlations. As shown in Table 14, quiz scores were positively and significantly associated with all six dimensions (rs = 0.218–0.317, ps < 0.001). The strongest associations were observed for PATI (*r* = 0.317) and AFLA (*r* = 0.307), whereas PDCC showed the weakest association (*r* = 0.218). These findings indicate positive correspondence between self-reported technological competence and performance on the framework-aligned scenario-based judgment measure.

**Table 14:** Correlations between quiz score and technological competence constructs’ average scores.

Variable	*r*	*p*
PDCC_avg—quiz score	0.218	<0.001
AFLA_avg—quiz score	0.307	<0.001
TTSE_avg—quiz score	0.259	<0.001
ITTA_avg—quiz score	0.250	<0.001
PATI_avg—quiz score	0.317	<0.001
FDTU_avg—quiz score	0.253	<0.001

Exploratory domain-matched analyses were also conducted between each self-report subscale and its corresponding three-item scenario-domain score. All six theoretically matched associations were positive and statistically significant, ranging from *r* = 0.151 to *r* = 0.219, with all *p* < 0.001 (Table 15). PDCC was positively associated with the PDCC scenario domain, *r* = 0.151; AFLA with AFLA, *r* = 0.198; TTSE with TTSE, *r* = 0.212; ITTA with ITTA, *r* = 0.199; PATI with PATI, *r* = 0.219; and FDTU with FDTU, *r* = 0.172. These results demonstrate positive correspondence between each self-report domain and judgment on conceptually matched scenario items. The three-item domain scores were retained as exploratory indicators rather than interpreted as independent psychometric subscores.

**Table 15:** Exploratory domain-matched correlations between self-report subscales and corresponding scenario-based quiz domains.

Self-report subscale	Corresponding scenario domain	Pearson’s *r*	*p*
PDCC	PDCC	0.151	<0.001
AFLA	AFLA	0.198	<0.001
TTSE	TTSE	0.212	<0.001
ITTA	ITTA	0.199	<0.001
PATI	PATI	0.219	<0.001
FDTU	FDTU	0.172	<0.001

Each scenario-domain score comprised three dichotomously scored items. These correlations are reported as exploratory domain-matched associations; reliability was established for the complete 18-item quiz rather than for the six three-item domain scores.

Multiple linear regression was used to examine the simultaneous and unique concurrent associations of the six technological competence dimensions with quiz performance. The full model including PDCC, AFLA, TTSE, ITTA, PATI, and FDTU was statistically significant, *F*(6, 967) = 88.34, *p* < 0.001, explaining 35.4% of the variance in quiz scores (*R* = 0.595, *R*^2^ = 0.354, adjusted *R*^2^ = 0.350; Tables 16, 17). All predictors contributed significantly and positively to quiz performance (*ps* < 0.001). PATI showed the largest standardized coefficient (β = 0.265), followed by FDTU (β = 0.228), AFLA (β = 0.216), ITTA (β = 0.213), PDCC (β = 0.203), and TTSE (β = 0.188).

**Table 16:** Model summary and ANOVA for regression predicting quiz score.

Model	*R*	*R* ^2^	Adj. *R*^2^	RMSE	Δ*R*^2^	*F*(df1, df2)	*p*
M₀	0.000	0.000	0.000	4.805	—	—	—
M₁	0.595	0.354	0.350	3.874	0.354	88.34 (6, 967)	< 0.001

**Table 17:** Regression coefficients and collinearity statistics.

Predictor	*B*	SE	*β*	*t*	*p*	Tolerance	VIF
(Intercept)	−11.17	0.967	—	−11.55	<0.001	—	—
PDCC_avg	1.257	0.164	0.203	7.67	<0.001	0.956	1.046
AFLA_avg	1.216	0.151	0.216	8.04	<0.001	0.925	1.082
TTSE_avg	1.275	0.179	0.188	7.12	<0.001	0.954	1.048
ITTA_avg	1.130	0.139	0.213	8.15	<0.001	0.982	1.019
PATI_avg	1.344	0.137	0.265	9.84	<0.001	0.920	1.087
FDTU_avg	1.219	0.140	0.228	8.73	<0.001	0.981	1.020

Collinearity diagnostics indicated no multicollinearity concerns, with tolerance values ranging from 0.920 to 0.982 and VIF values from 1.02 to 1.09 (Table 17). Overall, the regression findings indicate that the six dimensions contributed complementary concurrent information about performance on the framework-aligned scenario-based judgment measure. These coefficients should not be interpreted as prospective prediction or causal effects.

Overall, the six technological competence dimensions were meaningfully associated with quiz performance. However, because the quiz was administered concurrently, was conceptually aligned with the same six domains, and did not directly assess classroom practice, the findings should be interpreted as preliminary framework-aligned concurrent evidence rather than independent criterion validity, behavioral validation, or prospective predictive validity.

### 3.7 Reliability

Internal consistency reliability was evaluated using McDonald’s omega (*ω*) and Cronbach’s alpha (*α*) for both the pilot sample (EFA) and the main sample (CFA). As shown in Table 18, reliability coefficients were consistently high across all constructs. In the pilot sample, ω values ranged from 0.814 to 0.895 and *α* values ranged from 0.818 to 0.892, indicating good to excellent internal consistency. In the main sample, *ω* values ranged from 0.804 to 0.878 and *α* values ranged from 0.811 to 0.877, similarly demonstrating strong reliability across all factors. Overall 33-item coefficients were *ω* = 0.919 and *α* = 0.805 in the pilot sample and *ω* = 0.889 and *α* = 0.779 in the main sample. These coefficients are reported for descriptive completeness because all items were administered within one instrument; they should not be interpreted as evidence of unidimensionality or as justification for calculating an undifferentiated total score. Primary interpretation should remain at the six-subscale level. Across both samples, all subscales exceeded the commonly accepted threshold of 0.70, supporting the internal consistency of the measurement model (Table 18).

**Table 18:** Internal consistency reliability estimates for the pilot (EFA) and main (CFA) samples.

Factor	*ω* (EFA)	*α* (EFA)	*ω* (CFA)	*α* (CFA)
FDTU	0.895	0.892	0.878	0.877
PDCC	0.871	0.881	0.858	0.866
AFLA	0.877	0.876	0.861	0.850
PATI	0.884	0.888	0.865	0.863
ITTA	0.887	0.878	0.870	0.861
TTSE	0.814	0.818	0.804	0.811
Total	0.919	0.805	0.889	0.779

### 3.8 Construct means (retained-item scoring): EFA vs. CFA

Construct scores were calculated using only the items retained from the exploratory factor analysis to ensure consistency between the exploratory and confirmatory phases. Across both samples, ITTA showed the highest mean levels, followed by FDTU, AFLA, PATI, PDCC, and TTSE (Table 19). Mean levels were highly similar across samples. In the pilot sample, ITTA showed the highest mean (M = 3.57, SD = 0.90), followed by FDTU (M = 3.49, SD = 0.91), AFLA (M = 3.41, SD = 0.85), PATI (M = 3.35, SD = 0.95), PDCC (M = 1.99, SD = 0.76), and TTSE (M = 1.87, SD = 0.66). A highly similar pattern was observed in the main sample: ITTA (M = 3.53, SD = 0.90), FDTU (M = 3.46, SD = 0.90), AFLA (M = 3.37, SD = 0.85), PATI (M = 3.33, SD = 0.95), PDCC (M = 2.03, SD = 0.78), and TTSE (M = 1.94, SD = 0.71). Welch’s *t* tests indicated no statistically significant differences between samples for any construct (*ps* ≥ 0.085), and effect sizes were negligible (|*d*| ≤ 0.09). Similarly, overall scores were nearly identical across samples (EFA: M = 2.95, SD = 0.39; CFA: M = 2.94, SD = 0.38), *t* = 0.22, *p* = 0.827, *d* = 0.01. Overall, these findings indicate closely comparable construct means across the pilot and main samples, with no evidence of substantial mean-level differences on the variables examined.

**Table 19:** Construct means (retained-item scoring): EFA vs. CFA.

Scale/factor	Pilot (EFA) M (SD)	Main (CFA) M (SD)	Test statistic
FDTU	3.49 (0.91)	3.46 (0.90)	*t* = 0.51, *p* = 0.613, *d* = 0.03
PDCC	1.99 (0.76)	2.03 (0.78)	*t* = −1.06, *p* = 0.287, *d* = −0.06
AFLA	3.41 (0.85)	3.37 (0.85)	*t* = 0.91, *p* = 0.365, *d* = 0.05
PATI	3.35 (0.95)	3.33 (0.95)	*t* = 0.39, *p* = 0.695, *d* = 0.02
ITTA	3.57 (0.90)	3.53 (0.90)	*t* = 0.95, *p* = 0.342, *d* = 0.05
TTSE	1.87 (0.66)	1.94 (0.71)	*t* = −1.72, *p* = 0.085, *d* = −0.09
Overall	2.95 (0.39)	2.94 (0.38)	*t* = 0.22, *p* = 0.827, *d* = 0.01

### 3.9 Gender-stratified results

Following evidence of threshold invariance, construct means were examined descriptively by gender (Table 20). In the pilot sample, females showed slightly higher mean scores on FDTU, PDCC, and PATI, whereas males showed higher mean scores on AFLA, ITTA, and TTSE. The largest pilot-sample difference was observed for TTSE. In the main sample, females showed slightly higher mean scores on FDTU, PDCC, AFLA, PATI, and the overall retained-item average, whereas male scores were marginally higher for ITTA and TTSE. These results are descriptive and should be interpreted together with the measurement-invariance findings.

**Table 20:** Construct means (retained-item scoring) by gender.

Scale	Pilot male	Pilot female	Main male	Main female
FDTU	3.45 (0.96)	3.51 (0.88)	3.44 (0.93)	3.48 (0.88)
PDCC	1.95 (0.72)	2.01 (0.78)	1.95 (0.71)	2.07 (0.81)
AFLA	3.44 (0.83)	3.40 (0.86)	3.34 (0.84)	3.39 (0.86)
PATI	3.35 (0.92)	3.36 (0.98)	3.28 (0.91)	3.36 (0.96)
ITTA	3.63 (0.92)	3.54 (0.88)	3.53 (0.91)	3.52 (0.90)
TTSE	1.99 (0.69)	1.81 (0.63)	1.95 (0.68)	1.93 (0.73)
Overall	2.97 (0.39)	2.94 (0.39)	2.92 (0.34)	2.96 (0.41)

### 3.10 Item-level descriptive statistics

Item-level descriptive statistics were highly similar across the pilot and main samples (Table 21). In the pilot sample, item means ranged from 1.74 to 4.01, whereas in the main sample they ranged from 1.82 to 3.95. Across both samples, the highest means were observed for ITTA06 and FDTU05, whereas the lowest means were observed for TTSE items (particularly TTSE06 and TTSE04). Standard deviations ranged from 0.77 to 1.28 in the pilot sample and from 0.89 to 1.24 in the main sample, indicating adequate response variability across retained items.

**Table 21:** Item-level means and standard deviations for retained items across pilot (EFA) and main (CFA) samples.

Construct	Item	Pilot (EFA) M (SD)	Main (CFA) M (SD)
FDTU	FDTU01	3.43 (1.14)	3.40 (1.19)
	FDTU02	3.28 (1.19)	3.25 (1.16)
	FDTU03	3.35 (1.19)	3.32 (1.17)
	FDTU04	3.74 (1.07)	3.72 (1.11)
	FDTU05	3.82 (0.97)	3.80 (1.05)
	FDTU06	3.30 (1.17)	3.29 (1.16)
PDCC	PDCC01	1.89 (0.96)	1.95 (0.98)
	PDCC02	2.29 (0.90)	2.33 (1.03)
	PDCC03	2.03 (0.99)	2.09 (1.02)
	PDCC05	1.89 (0.95)	1.90 (0.98)
	PDCC06	1.89 (0.97)	1.93 (0.99)
	PDCC08	1.93 (0.96)	1.99 (1.01)
AFLA	AFLA03	3.04 (1.15)	2.96 (1.19)
	AFLA04	3.16 (1.20)	3.13 (1.18)
	AFLA05	3.82 (0.79)	3.79 (0.92)
	AFLA06	3.66 (1.05)	3.64 (1.12)
	AFLA07	3.48 (1.07)	3.44 (1.12)
	AFLA08	3.31 (1.14)	3.25 (1.21)
PATI	PATI01	3.20 (1.20)	3.14 (1.22)
	PATI03	3.23 (1.28)	3.18 (1.24)
	PATI04	3.49 (1.09)	3.49 (1.13)
	PATI06	3.46 (1.09)	3.45 (1.16)
	PATI07	3.40 (1.06)	3.41 (1.15)
ITTA	ITTA02	3.39 (1.15)	3.30 (1.17)
	ITTA03	3.70 (1.04)	3.65 (1.12)
	ITTA06	4.01 (0.95)	3.95 (1.02)
	ITTA07	3.42 (1.16)	3.36 (1.17)
	ITTA08	3.35 (1.17)	3.36 (1.15)
TTSE	TTSE01	2.02 (0.77)	2.09 (0.89)
	TTSE02	1.81 (0.86)	1.86 (0.92)
	TTSE03	2.02 (0.93)	2.07 (1.02)
	TTSE04	1.79 (0.87)	1.85 (0.93)
	TTSE06	1.74 (0.88)	1.82 (0.94)

## 4 Discussion

### 4.1 Summary of the main contribution

This study developed and validated a multidimensional measure of pre-service teachers’ technological competence for teaching by integrating TPACK, DigCompEdu, and technology-related self-efficacy into a single measurement framework. The findings support a six-factor structure consisting of Foundational Digital Tool Use, Professional Digital Communication and Collaboration, Assessment, Feedback, and Learning Analytics, Pedagogically Aligned Technology Integration, Instructional Technology Troubleshooting and Adaptability, and Teaching-Related Technology Self-Efficacy. This structure reflects a broad view of technological competence that extends beyond basic digital skill and beyond a narrow focus on technology knowledge alone.

The results provide empirical support for interpreting technological competence for teaching as multidimensional rather than as a single homogeneous general ability. The six dimensions represent operational, professional, pedagogical, adaptive, assessment-oriented, and belief-based components of technology use in teaching. Some dimensions showed meaningful associations, whereas several others were weakly or negligibly related, supporting primary interpretation at the subscale level.

This finding is consistent with TPACK research emphasizing the integration of technology, pedagogy, and content (Ifinedo et al., 2020; Syamdianita and Cahyono, 2021; Wu et al., 2015), while also extending that literature by incorporating DigCompEdu-informed professional and assessment dimensions (Sofwan et al., 2024) and technology-related self-efficacy (Giles and Kent, 2016; Pradita et al., 2023; Sridana et al., 2024).

### 4.2 Novelty of the scale

The main contribution of the present scale lies in its integration of components that existing instruments generally assess separately. Many established measures focus primarily on TPACK-related knowledge domains, particularly teachers’ perceived knowledge of technology, pedagogy, content, and their intersections (Anthony et al., 2021; Nurwahidah et al., 2023; Schmidt et al., 2009; Syamdianita and Cahyono, 2021). The TPACK survey developed by Schmidt et al. (2009), for example, assesses seven knowledge domains and provides valuable information about pre-service teachers’ perceived technological, pedagogical, and content-related knowledge. However, TPACK-oriented measures typically place less emphasis on broader professional digital competence, technology-supported assessment, troubleshooting, adaptability, and confidence. The present scale extends this measurement tradition by integrating knowledge-related, professional, adaptive, and belief-based components within a single multidimensional instrument.

This broader perspective is strengthened by the explicit incorporation of DigCompEdu-oriented professional and assessment dimensions. The DigCompEdu Check-In assesses six areas of educators’ digital competence, including professional engagement, digital resources, teaching and learning, assessment, learner empowerment, and facilitation of learners’ digital competence (Cabero-Almenara et al., 2020). Drawing on this broader conception of educator digital competence, the present scale includes Professional Digital Communication and Collaboration (PDCC) and Assessment, Feedback, and Learning Analytics (AFLA) as distinct dimensions. PDCC captures the use of digital technologies in institutional, peer, and professional learning contexts, whereas AFLA addresses the use of digital tools and evidence to monitor learning, provide feedback, and inform instructional decisions. Their inclusion extends the scale beyond knowledge-centered technology integration and provides a more comprehensive representation of the professional and assessment-related demands of technology-supported teaching.

A further contribution is the inclusion of Teaching-Related Technology Self-Efficacy (TTSE) as a separate dimension. Technology-integration self-efficacy measures, such as that developed by Wang et al. (2004), focus specifically on individuals’ confidence in their capability to integrate technology into instruction. Previous research also links technology-related self-efficacy with persistence, technology adoption, and instructional behavior (Giles and Kent, 2016; Pradita et al., 2023; Sridana et al., 2024). Incorporating TTSE therefore recognizes that technological competence for teaching cannot be represented fully by knowledge and reported practice alone. Pre-service teachers must also believe that they can select, adapt, troubleshoot, and sustain technology-supported instructional practices when difficulties arise.

The present 33-item scale combines these complementary theoretical traditions while retaining six empirically distinguishable subscales. Foundational Digital Tool Use (FDTU) captures operational capability; PDCC represents Professional Digital Communication and Collaboration; AFLA addresses assessment, feedback, and evidence use; Pedagogically Aligned Technology Integration (PATI) represents the alignment of technology with instructional goals; Instructional Technology Troubleshooting and Adaptability (ITTA) captures adaptive responses to technological and contextual constraints; and TTSE represents capability beliefs related to technology-supported teaching. The resulting structure therefore extends beyond knowledge-centered TPACK measurement, broad digital-competence self-reflection, and stand-alone self-efficacy assessment by bringing these complementary components together within one instrument designed specifically for pre-service teachers.

An additional contribution is the scale’s subscale-level profile structure. Rather than reducing technological competence to a single general score, the six subscales provide differentiated information about comparatively stronger and weaker areas of self-reported competence. For example, lower FDTU scores indicate comparatively lower self-reported competence in Foundational Digital Tool Use, lower AFLA scores indicate comparatively lower self-reported competence in Assessment, Feedback, and Learning Analytics, and lower TTSE scores indicate comparatively lower Teaching-Related Technology Self-Efficacy. This profile-based interpretation is consistent with the empirical differentiation observed among the six dimensions and allows the instrument to represent operational, professional, assessment-related, pedagogical, adaptive, and belief-based aspects of technological competence without requiring them to function as interchangeable indicators of a homogeneous general factor. These profiles may support research and formative program-level evaluation, but they should not be interpreted in isolation as diagnostic evidence that particular individuals require intervention.

### 4.3 Interpretation of the six-factor structure

The six-factor structure is theoretically meaningful and empirically stable. Foundational Digital Tool Use represents the operational base of technology-supported teaching. This dimension aligns with research showing that pre-service teachers often begin with common tools such as presentation software, learning platforms, mobile devices, and classroom applications (Abubakir and Alshaboul, 2023; Imania et al., 2022). Operational skill is necessary, but it is not sufficient for effective technology integration.

Professional Digital Communication and Collaboration reflects the social and professional dimension of digital competence. DigCompEdu emphasizes that educators use digital tools not only for instruction, but also for communication, collaboration, professional exchange, and participation in digital learning environments (Sofwan et al., 2024). The inclusion of this factor strengthens the scale because teaching increasingly requires interaction through digital platforms, learning-management systems, and professional networks.

Assessment, Feedback, and Learning Analytics emerged as a distinct dimension, supporting the claim that assessment-related technology use deserves separate measurement. This finding is important because prior studies report that pre-service teachers often show weaknesses in technology-supported assessment, feedback, and evidence use (Abubakir and Alshaboul, 2023; Choi and Chung, 2025). The distinctiveness of this factor shows that assessment-oriented competence is not reducible to general tool use or general pedagogical integration.

Pedagogically aligned technology integration represents the core TPACK-related dimension of the scale. This factor reflects the ability to select and use technology in ways that support instructional goals, content representation, and student learning. This result is consistent with TPACK literature emphasizing that effective technology use depends on meaningful alignment among technology, pedagogy, and content rather than superficial tool adoption (Ifinedo et al., 2020; Tan et al., 2023; Wu et al., 2015).

Instructional technology troubleshooting and adaptability reflects the flexible and problem-solving nature of technology-rich teaching. Technology integration often involves technical disruptions, changing classroom conditions, and the need to revise instruction during implementation. This factor therefore adds an applied and adaptive component to the measurement of technological competence. It connects to research showing that successful technology integration requires reflection, revision, and adjustment in authentic teaching contexts (Abubakir and Alshaboul, 2023; Wardani et al., 2020).

Teaching-Related Technology Self-Efficacy represents confidence in using technology for instructional purposes. Its inclusion is theoretically justified because self-efficacy influences persistence, willingness to experiment, and sustained engagement with technology integration (Giles and Kent, 2016; Pradita et al., 2023; Sridana et al., 2024). The distinctness of this factor supports treating technology-related confidence as conceptually and empirically distinguishable from operational skill and pedagogical knowledge.

### 4.4 Psychometric evidence and construct validity

The exploratory and confirmatory results support the multidimensional structure of the scale. The EFA identified a six-factor structure with clear theoretical interpretability, and the CFA confirmed that the same structure fit the main sample well. The use of independent samples for EFA and CFA strengthens the validation design because the factor structure was not tested on the same data used for item reduction.

The CFA fit indices supported the proposed model, and the squared standardized loadings, AVE values, and HTMT ratios provided evidence concerning convergent validity and empirical differentiation among the dimensions. The factor relationships were heterogeneous: some dimensions showed modest positive associations, whereas several relationships were weak, negligible, or slightly negative. This pattern supports a deliberately multidimensional interpretation. In the present framework, the six dimensions are grouped because they represent theoretically relevant aspects of technology-supported teaching, rather than because they constitute interchangeable manifestations of a single homogeneous latent ability. The weak and near-zero relationships demonstrate substantial empirical distinctiveness among particular dimensions and argue against interpretation through an undifferentiated overall score or a required higher-order factor. Accordingly, technological competence for teaching, as operationalized here, is best interpreted as a profile of six conceptually related but empirically distinguishable subscale scores.

Reliability evidence also supported the scale. McDonald’s omega and Cronbach’s alpha values exceeded commonly accepted thresholds across the subscales in both the pilot and main samples. This consistency across samples indicates that the retained items functioned reliably across the exploratory and confirmatory phases. TTSE nevertheless showed the lowest reliability coefficients among the six subscales and the lowest AVE (0.520), although these values remained within the criteria used in the present study. TTSE is theoretically distinguished from the other dimensions because it represents beliefs about capability rather than direct evidence of knowledge, skill, practice, or demonstrated performance. The present data do not identify the reason for its comparatively lower psychometric coefficients. The TTSE findings therefore warrant continued evaluation in subsequent validation studies.

Measurement invariance across gender provides additional support for the scale. The configural, metric, and scalar models showed acceptable fit and negligible changes in approximate fit indices. These findings indicate that the scale measured the same constructs across male and female pre-service teachers, supporting meaningful gender-based comparisons in this sample.

### 4.5 Interpretation of construct means

The descriptive findings showed higher mean levels for Instructional Technology Troubleshooting and Adaptability and Foundational Digital Tool Use, whereas lower mean levels appeared for Professional Digital Communication and Collaboration and Teaching-Related Technology Self-Efficacy. This pattern is informative for teacher education. It suggests that pre-service teachers in the sample reported comparatively higher technological competence in the operational and adaptive dimensions than in teaching-related self-efficacy and Professional Digital Communication and Collaboration.

This uneven profile is consistent with previous research showing that pre-service teachers often develop familiarity with tools before developing deeper pedagogical, assessment-oriented, and confidence-based competence (Abubakir and Alshaboul, 2023; Choi and Chung, 2025; Hsu et al., 2023; Uygun et al., 2023). The results therefore support the need for teacher-education programs to move beyond tool exposure and toward structured opportunities for technology-integrated lesson design, digital assessment, collaborative practice, troubleshooting, and supervised implementation.

The comparatively low self-efficacy scores are noteworthy, but they should not be interpreted as direct evidence that individual participants require intervention. At the program level, the pattern suggests that teacher-education curricula may benefit from repeated, supported opportunities for technology-integrated practice, modeling, feedback, and practicum-based implementation.

This interpretation aligns with self-efficacy research emphasizing the role of mastery experiences and supported practice in strengthening confidence for technology integration (Giles and Kent, 2016; Pradita et al., 2023; Sridana et al., 2024).

### 4.6 Concurrent associations with scenario-based judgment

All six technological competence dimensions were positively associated with performance on the scenario-based judgment measure. The strongest associations were observed for Pedagogically Aligned Technology Integration and Assessment, Feedback, and Learning Analytics, which is consistent with the applied nature of the scenarios, particularly their emphasis on instructional alignment, assessment, feedback, and technology use. In the simultaneous regression model, all six dimensions also showed significant positive unique associations with quiz performance. PATI had the largest standardized coefficient, followed by FDTU, AFLA, ITTA, PDCC, and TTSE. Taken together, these findings indicate that the six dimensions contributed complementary information about concurrent scenario-based judgment despite several weak inter-factor relationships.

The domain-matched results also clarify the level at which the scenario measure should be interpreted. Although all six matched associations were positive and statistically significant, matched-domain coefficients were not uniformly larger than cross-domain associations. This pattern reflects the integrated nature of situational judgment. Contextualized scenarios can draw simultaneously on multiple competencies, and empirical SJT research has demonstrated multidimensional structures and cross-factor loadings when successful judgment requires several related competencies (Black et al., 2021). Construct-driven SJT research likewise shows that achieving sharply differentiated measurement of specific attributes requires scenarios specifically designed to isolate those constructs (Mielke et al., 2022). In the present quiz, effective responses to technology-supported teaching problems require combinations of Technological Knowledge, pedagogical alignment, assessment reasoning, adaptability, professional judgment, and persistence. Combined with the limited three-item coverage of each domain, this structure supports interpretation of the quiz primarily as an overall framework-aligned technological-pedagogical judgment measure rather than as six independent criterion scales (Haberman, 2008; Sinharay et al., 2011). Accordingly, the total quiz score served as the concurrent criterion, and the three-item domain scores were not treated as established psychometric subscores.

These findings should nevertheless be interpreted cautiously. The quiz was developed within the same research framework, was conceptually aligned with the six technological competence domains, and was administered during the same session as the self-report scale. Although the two measures used different response formats, their shared conceptual framework and administration context introduce shared method variance and context-related influences as alternative explanations for part of the observed associations. The present design does not separate these influences from construct-related correspondence. The observed relationships therefore constitute preliminary framework-aligned concurrent evidence rather than validation against an independent external criterion, evidence of actual classroom behavior, or prospective predictive validity. Future studies should evaluate the scale against independently developed performance measures administered separately from the self-report instrument, as well as classroom observations, practicum evaluations, instructional artifacts, and longitudinal teaching outcomes.

### 4.7 Implications for teacher education

The scale has practical value for teacher-education research and formative program evaluation because it provides separate information across six technological competence domains. Given the weak or negligible relationships among several dimensions, a single total score would obscure important differences among the subscales. The resulting profiles can identify areas of comparatively higher or lower self-reported technological competence that may warrant further investigation at the individual or program level.

Teacher educators should use the subscale profiles as one source of formative information for curriculum planning and program evaluation. For example, comparatively lower scores in Foundational Digital Tool Use may identify an area warranting further examination of operational preparation, whereas comparatively lower scores in Assessment, Feedback, and Learning Analytics may indicate an area in which additional learning opportunities could be considered. Similarly, lower scores in Pedagogically Aligned Technology Integration, troubleshooting and adaptability, or Teaching-Related Technology Self-Efficacy may help identify domains for further formative investigation. These interpretations should be considered alongside other evidence and should not be used to infer individual competence deficits or determine intervention needs from scale scores alone.

Future longitudinal research should examine whether the scale is sensitive to change following coursework, practicum experiences, workshops, or technology-integration interventions. Until sensitivity to change has been established, pre-post differences should not automatically be interpreted as evidence of program effectiveness.

### 4.8 Theoretical implications

The findings contribute to theory by showing that TPACK, DigCompEdu, and self-efficacy represent complementary rather than competing perspectives. TPACK explains the pedagogical alignment of technology, content, and instruction. DigCompEdu broadens the construct by adding professional communication, digital resources, teaching, learning, and assessment dimensions. Self-efficacy adds a motivational and belief-based component associated with persistence and sustained technology use. The present scale integrates these perspectives within a multidimensional measurement framework while preserving their empirical distinctiveness.

This integrated structure is important because teacher technological competence is often discussed in fragmented ways. Some studies emphasize knowledge, others emphasize digital skills, and others emphasize confidence or attitudes. The present findings support a more comprehensive view in which technological competence includes what pre-service teachers know, what they do, how they adapt, how they assess, how they communicate professionally, and how confident they feel in technology-supported teaching.

### 4.9 Limitations and future research

Several limitations should be considered when interpreting the findings.

- First, the study was conducted with pre-service teachers from a single public university in Türkiye. Although the sample size was large and the use of nonoverlapping pilot and main samples strengthened the validation design, both cohorts were recruited within the same institutional setting and both data collections occurred during April 2026. The demographic comparisons indicated similarity on the variables examined, but the study did not evaluate cohort differences in instructional exposure, coursework, instructors, or other institutional conditions. The influence of unmeasured cohort-specific conditions therefore remains unresolved. Broader generalization and further cross-validation require evidence from different institutions, regions, teacher-education programs, and independently recruited cohorts.
- Second, the scale is based on self-report data. Self-report instruments are useful for assessing perceived competence, confidence, readiness, and reported practices, but they are influenced by social desirability, response style, and participants’ self-awareness. Accordingly, the subscale scores do not constitute direct observations of teaching performance. Future studies should examine the scale alongside observational data, teaching-performance tasks, practicum evaluations, lesson-plan analyses, and digital portfolios.
- Third, the item-reduction process resulted in unequal numbers of retained items across the six dimensions. Although each intended dimension remained represented in the final 33-item solution, FDTU, PDCC, and AFLA retained six items each, whereas PATI, ITTA, and TTSE retained five items each. The present factorial results support the retained structure, but they do not establish that every facet represented in the larger initial item pool was preserved to the same extent. Future studies should therefore continue to examine the content coverage of the retained subscales.
- Fourth, the scenario-based quiz provided a response format different from the self-report scale, but it was developed within the same conceptual framework and administered concurrently. It therefore did not constitute an independent external criterion or a test of prospective prediction. Shared conceptual content and same-session administration introduce shared method variance and administration-context effects as alternative explanations for part of the observed associations. The present design does not quantify the contribution of these influences. In addition, although the quiz included three items corresponding to each conceptual domain, the present study established reliability for the full 18-item quiz rather than for six separate three-item domain subscores. The reported associations therefore concern overall framework-aligned judgment rather than established domain-specific external criteria. Future research should examine the scale against independently developed and independently administered performance measures with sufficient domain-level item coverage, classroom observations, technology-integrated teaching tasks, practicum ratings, instructional artifacts, and longitudinal outcomes.
- Fifth, the English and Turkish item versions were prepared in parallel and reviewed for clarity, linguistic appropriateness, and correspondence of meaning. However, the present validation did not provide quantitative evidence of cross-language measurement equivalence. Because the instrument was administered in Turkish in the current study, direct evidence regarding psychometric equivalence between the English and Turkish versions remains unavailable. Future research using both language versions should examine cross-language equivalence directly.
- Sixth, TTSE showed the lowest reliability coefficients among the six subscales and the lowest AVE, although its values remained within the criteria applied in the present study. TTSE represents a belief-based dimension that is conceptually distinct from direct knowledge, skill, reported practice, and demonstrated performance. The present data do not identify the source of its comparatively lower psychometric coefficients. Continued validation should therefore examine the TTSE dimension across independent samples and contexts.
- Seventh, the study used a cross-sectional design. The findings show relationships among constructs at one point in time but do not establish developmental change across teacher preparation. Reliability was evaluated through internal-consistency coefficients rather than repeated administration; therefore, the temporal stability of the six subscale scores remains untested. Future research should evaluate test–retest reliability and use longitudinal designs to distinguish score stability from change associated with coursework, practicum experience, or targeted technology-integration interventions.
- Finally, measurement invariance was examined only across gender. Future research should test invariance across year of study, academic program, prior technology experience, institutional context, language, and cultural setting. Such evidence would strengthen the basis for group comparison, formative interpretation, and program-level evaluation.

## 5 Conclusion

This study developed and validated a multidimensional scale for assessing pre-service teachers’ technological competence for teaching. The instrument was grounded in complementary elements of TPACK, DigCompEdu, and technology-related self-efficacy and was evaluated using independent exploratory and confirmatory samples. The resulting 33-item scale represents six dimensions: Foundational Digital Tool Use, Professional Digital Communication and Collaboration, Assessment, Feedback, and Learning Analytics, Pedagogically Aligned Technology Integration, Instructional Technology Troubleshooting and Adaptability, and Teaching-Related Technology Self-Efficacy.

The findings support a multidimensional rather than unitary interpretation of technological competence for teaching. Exploratory factor analysis identified a six-factor structure, and confirmatory factor analysis supported the same structure in the independent main sample. The subscales demonstrated satisfactory internal consistency, convergent validity, empirical differentiation, and gender measurement invariance. Several inter-factor relationships were weak or negligible, indicating that the six dimensions should not be treated as interchangeable indicators of a single homogeneous general factor. Accordingly, primary interpretation should focus on the six subscale scores rather than an undifferentiated total score.

The six dimensions also showed positive concurrent associations with performance on the scenario-based technological-pedagogical judgment measure. Pedagogically Aligned Technology Integration and Assessment, Feedback, and Learning Analytics showed the strongest associations with quiz performance, while all six dimensions contributed significant unique information in the simultaneous regression model. These findings provide preliminary evidence that self-reported technological competence corresponds with judgment in conceptually aligned teaching scenarios. However, because the quiz was administered concurrently and was conceptually aligned with the same technological competence domains, these associations should not be interpreted as independent criterion validity, direct evidence of classroom behavior, causal effects, or prospective predictive validity.

The scale is intended primarily for subscale-level research, formative interpretation, and program-level evaluation. Its profile structure can be used to describe comparatively stronger and weaker areas of self-reported technological competence across operational tool use, Professional Digital Communication and Collaboration, digital assessment and evidence use, pedagogically aligned integration, adaptive problem solving, and Teaching-Related Technology Self-Efficacy. Such profiles may help identify domains warranting further investigation or instructional attention, but individual scores should not by themselves be treated as diagnostic evidence of competence deficits or as sufficient grounds for high-stakes decisions.

The present findings should be regarded as initial validation evidence rather than final confirmation of universal applicability. Both samples were drawn from one public university in Türkiye, and the scale relies on self-report rather than direct observation of teaching performance. Further validation is therefore needed across institutions, teacher-education programs, cultural contexts, and levels of prior technology experience. Future research should also examine the scale in relation to independently developed performance measures, classroom observations, practicum evaluations, instructional artifacts, digital portfolios, and longitudinal teaching outcomes.

Overall, the study provides initial psychometric support for a six-dimensional self-report measure of technological competence for teaching. By integrating operational, professional, assessment-related, pedagogical, adaptive, and belief-based dimensions within a single measurement framework while preserving their empirical distinctiveness, the scale offers a structured approach to examining technological competence among pre-service teachers. Further behavioral, longitudinal, and cross-context validation will be important before broader or higher-stakes interpretations of the scores are warranted.

## Data Availability

The datasets supporting this study, including detailed demographic statistics, content validity ratings, and the full survey and quiz instruments, are available on Zenodo at https://doi.org/10.5281/zenodo.20772820. The appendices are provided in the Supplementary File.

## References

[ref1] Abubakir H. Alshaboul Y. (2023). Unravelling EFL teachers’ mastery of TPACK: technological pedagogical and content knowledge in writing classes. Heliyon 9:Article e17348:e17348. doi: 10.1016/j.heliyon.2023.e17348, 37441392 PMC10333461

[ref2] Anthony B. Kamaludin A. Romli A. (2021). Predicting academic staffs’ behaviour intention and actual use of blended learning in higher education: model development and validation. Technol. Knowl. Learn. 28, 1223–1269. doi: 10.1007/s10758-021-09579-2

[ref3] Bakar N. S. A. Maat S. M. Rosli R. (2020). Mathematics teacher’s self-efficacy of technology integration and technological pedagogical content knowledge. J. Math. Educ. 11, 259–276. doi: 10.22342/jme.11.2.10818.259-276

[ref4] Bandura A. (1977). Self-efficacy: toward a unifying theory of behavioral change. Psychol. Rev. 84, 191–215. doi: 10.1037/0033-295X.84.2.191 847061

[ref5] Black E. W. Schrock B. Prewett M. S. Blue A. V. (2021). Design of a situational judgment test for preclinical interprofessional collaboration. Acad. Med. 96, 992–996. doi: 10.1097/ACM.0000000000004117, 33830952

[ref6] Cabero-Almenara J. Gutiérrez-Castillo J.-J. Palacios-Rodríguez A. Barroso-Osuna J. (2020). Development of the teacher digital competence validation of DigCompEdu check-in questionnaire in the university context of Andalusia (Spain). Sustainability 12:6094. doi: 10.3390/su12156094

[ref7] Chang C.-F. Annisa N. Chen K.-Z. (2024). Pre-service teacher professional education program (PPG) and Indonesian science teachers’ TPACK development: a career-path comparative study. Educ. Inf. Technol. 30, 8689–8711. doi: 10.1007/s10639-024-13160-6

[ref8] Choi L. J. Chung S. J. (2025). Preparing technologically competent EFL teachers: a TPACK-based approach. SAGE Open 15:21582440251367827. doi: 10.1177/21582440251367827

[ref9] Costello A. B. Osborne J. W. (2005). Best practices in exploratory factor analysis: four recommendations for getting the most from your analysis. Pract. Assess. Res. Eval. 10, 1–9. doi: 10.7275/jyj1-4868

[ref10] Dağgöl G. D. (2022). Melting the content, pedagogy and technology in the same pot: insights into EFL instructors’ TPACK perceptions in digital era. IJELTAL 6:307. doi: 10.21093/ijeltal.v6i2.1096

[ref11] Fabrigar L. R. Wegener D. T. MacCallum R. C. Strahan E. J. (1999). Evaluating the use of exploratory factor analysis in psychological research. Psychol. Methods 4, 272–299. doi: 10.1037/1082-989X.4.3.272

[ref12] Giles R. M. Kent A. M. (2016). An investigation of preservice teachers’ self-efficacy for teaching with technology. Asian Educ. Stud. 1, 32–40. doi: 10.20849/aes.v1i1.19

[ref13] Gromik N. Litz D. Liu B. (2024). Technology, pedagogy, and content knowledge: an Australian case study. Educ. Sci. 14:Article 37. doi: 10.3390/educsci14010037

[ref14] Haberman S. J. (2008). When can subscores have value? J. Educ. Behav. Stat. 33, 204–229. doi: 10.3102/1076998607302636

[ref15] Hinkin T. R. (1998). A brief tutorial on the development of measures for use in survey questionnaires. Organ. Res. Methods 1, 104–121. doi: 10.1177/109442819800100106

[ref16] Hsu H.-P. Cheah Y. H. Hughes J. E. (2023). A case study of a secondary biology teacher’s pedagogical reasoning and action with augmented reality technology. Educ. Sci. 13:1080. doi: 10.3390/educsci13111080

[ref17] Huang C. Xu W. Shen K. (2025). Mapping the landscape of TPACK in English language learning. Int. J. Web-Based Learn. Teach. Technol. 20, 1–18. doi: 10.4018/ijwltt.373233

[ref18] Ifinedo E. Rikala J. Hämäläinen T. (2020). Factors affecting Nigerian teacher educators’ technology integration: considering characteristics, knowledge constructs, ICT practices and beliefs. Comput. Educ. 146:Article 103760. doi: 10.1016/j.compedu.2019.103760

[ref19] Imania S. Anugerahwati M. Tresnadewi S. (2022). Unpacking EFL teachers’ TPACK thru the distance education. J. Pendidik. Teori Penelitian Pengembangan 7:51. doi: 10.17977/jptpp.v7i2.15188

[ref20] Mielke I. Breil S. M. Amelung D. Espe L. Knorr M. (2022). Assessing distinguishable social skills in medical admission: does construct-driven development solve validity issues of situational judgment tests? BMC Med. Educ. 22:293. doi: 10.1186/s12909-022-03305-x, 35440029 PMC9020047

[ref9021] Mishra P. Koehler M. J. (2006). Technological pedagogical content knowledge: a framework for teacher knowledge. Teach. Coll. Rec. 108, 1017–1054. doi: 10.1111/j.1467-9620.2006.00684.x

[ref22] Nepembe V. Simuja C. (2023). Instructors’ perspectives of TPACK in a vocational training classroom in Namibia. J. Vocat. Adult Contin. Educ. Train. 6:18. doi: 10.14426/jovacet.v6i1.315

[ref23] Nurwahidah N. Sulfasyah S. Rukli R. (2023). Investigating grade five teachers’ integration of technology in teaching reading comprehension using the TPACK framework. J. Lang. Teach. Res. 14, 927–932. doi: 10.17507/jltr.1404.09

[ref24] Pradita L. Mardiana W. Mustpa D. (2023). Pre-service EFL teachers’ experience in integrating TPACK during teaching practice program. UC J. 4, 51–63. doi: 10.24071/uc.v4i1.5613

[ref25] Redecker C. (2017). In European Framework for the digital Competence of Educators: DigCompEdu, ed. Punie Y. (EUR 28775 EN) (Luxembourg City, Luxembourg: Publications Office of the European Union).

[ref26] Schmidt D. A. Baran E. Thompson A. D. Mishra P. Koehler M. J. Shin T. S. (2009). Technological pedagogical content knowledge (TPACK): the development and validation of an assessment instrument for preservice teachers. J. Res. Technol. Educ. 42, 123–149. doi: 10.1080/15391523.2009.10782544

[ref27] Sinharay S. Puhan G. Haberman S. J. (2011). An NCME instructional module on subscores. Educ. Meas. Issues Pract. 30, 29–40. doi: 10.1111/j.1745-3992.2011.00208.x

[ref28] Sofwan M. Yaakob M. F. M. Habibi A. (2024). Technological, pedagogical, and content knowledge for technology integration: a systematic literature review. Int. J. Eval. Res. Educ. 13:212. doi: 10.11591/ijere.v13i1.26643

[ref29] Sridana N. Novitasari D. Magfirah N. (2024). Analysis of preservice teachers’ ability to develop TPACK integrated learning tools. Griya J. Math. Educ. Appl. 4, 197–212. doi: 10.29303/griya.v4i3.473

[ref30] Syamdianita S. Cahyono B. Y. (2021). The EFL pre-service teachers’ experiences and challenges in designing teaching materials using TPACK framework. Stud. Engl. Lang. Educ. 8, 561–577. doi: 10.24815/siele.v8i2.19202

[ref31] Tan L. Thomson R. Koh J. H. L. Chik A. (2023). Teaching multimodal literacies with digital technologies and augmented reality: a cluster analysis of Australian teachers’ TPACK. Sustainability 15:Article 10190. doi: 10.3390/su151310190, 30654563

[ref32] Uygun T. Şendur A. Dere R. Özçakır B. (2023). Development of TPACK with web 2.0 tools: design-based study. Eur. J. Sci. Math. Educ. 11, 445–465. doi: 10.30935/scimath/12907

[ref33] Wang L. Ertmer P. A. Newby T. J. (2004). Increasing preservice teachers’ self-efficacy beliefs for technology integration. J. Res. Technol. Educ. 36, 231–250. doi: 10.1080/15391523.2004.10782414

[ref34] Wardani E. N. Drajati N. A. Handayani E. I. P. (2020). Pre-service teacher experience in designing lesson using TPACK-21 CL in teaching reading for high school students. Edulangue 3, 29–48. doi: 10.20414/edulangue.v3i1.2094

[ref35] Worthington R. L. Whittaker T. A. (2006). Scale development research: a content analysis and recommendations for best practices. Counsel. Psychol. 34, 806–838. doi: 10.1177/0011000006288127

[ref36] Wu B. Hu Y. Gu X. Lim C. P. (2015). Professional development of new higher education teachers with information and communication technology in Shanghai. J. Educ. Comput. Res. 54, 531–562. doi: 10.1177/0735633115621922

