# Supplementary material for: A multidimensional measure of pre-service teachers’ perceived technological competence for teaching: integrating TPACK, DigCompEdu, and self-efficacy
Source: Front Psychol. 2026 Sep 8;17:1914635. doi: 10.3389/fpsyg.2026.1914635 (PMC13593813; doi:10.3389/fpsyg.2026.1914635)
Supplement: Supplementary file 1 [file Supplementary_File_1.pdf]

APPENDICES

Appendix A. Bilingual 90-Item Pool and Item-Retention Decisions Following Content Validation and EFA

Item	Item Text (English)	Item Text (Turkish)	Status	After EFA
FDTU1	I can navigate learning management systems independently.	Öğrenme yönetim sistemlerinde bağımsız olarak gezinebilirim.	Selected	Retained
FDTU2	I can use cloud storage platforms (e.g., Google Drive, OneDrive).	Bulut depolama platformlarını (ör. Google Drive, OneDrive) kullanabilirim.	Selected	Retained
FDTU3	I can use video conferencing tools without assistance.	Video konferans araçlarını yardım almadan kullanabilirim.	Selected	Retained
FDTU4	I can manage files and folders effectively on a computer.	Bilgisayardaki dosya ve klasörleri etkili bir şekilde düzenleyebilirim.	Selected	Retained
FDTU5	I can efficiently use word processing software for academic tasks.	Akademik çalışmalarımda kelime işlemci programlarını verimli bir şekilde kullanabilirim.	Selected	Retained
FDTU6	I can connect and configure digital devices (e.g., projector, printer).	Dijital cihazları (ör. projeksiyon cihazı, yazıcı) bağlayabilir ve yapılandırabilirim.	Selected	Retained
FDTU7	I can design multimedia presentations using digital tools.	Dijital araçları kullanarak çoklu ortam sunuları hazırlayabilirim.	Selected	Not Retained
FDTU8	I can install and update software applications when needed.	Gerektiğinde yazılım uygulamalarını yükleyebilir ve güncelleyebilirim.	Selected	Not Retained
FDTU9	I can create and edit spreadsheets for organizing data.	Verileri düzenlemek için elektronik tablolar oluşturabilir ve düzenleyebilirim.	Rejected	
FDTU10	I can use basic keyboard shortcuts to improve efficiency.	Daha verimli çalışmak için temel klavye kısayollarını kullanabilirim.	Rejected	
FDTU11	I can download and organize digital resources for teaching.	Öğretimde kullanacağım dijital kaynakları indirip düzenleyebilirim.	Rejected	
FDTU12	I can convert files between different formats (e.g., PDF, Word).	Dosyaları farklı biçimlere (ör. PDF, Word) dönüştürebilirim.	Rejected	
FDTU13	I can use collaborative document-editing platforms.	Ortak belge düzenleme platformlarını kullanabilirim.	Rejected	
FDTU14	I can adjust device settings to meet my needs.	Cihaz ayarlarını gereksinimlerime göre düzenleyebilirim.	Rejected	
FDTU15	I can protect my devices using basic security measures (e.g., passwords).	Temel güvenlik önlemlerini (ör. parola kullanma) uygulayarak cihazlarımı koruyabilirim.	Rejected	
PDCC1	I engage in online discussions about teaching practices.	Öğretim uygulamalarıyla ilgili çevrim içi tartışmalara katılırım.	Selected	Retained
PDCC2	I participate in online professional learning communities.	Çevrim içi mesleki öğrenme topluluklarına katılırım.	Selected	Retained
PDCC3	I maintain professional conduct in digital environments.	Dijital ortamlarda mesleki davranış kurallarına uygun hareket ederim.	Selected	Retained
PDCC4	I share teaching resources through digital networks.	Öğretim kaynaklarını dijital ağlar üzerinden paylaşırım.	Selected	Not Retained
PDCC5	I communicate effectively with students using digital tools.	Dijital araçları kullanarak öğrencilerle etkili iletişim kurarım.	Selected	Retained
PDCC6	I collaborate with others using digital platforms.	Dijital platformlar üzerinden başkalarıyla iş birliği yaparım.	Selected	Retained
PDCC7	I use digital platforms to coordinate group work.	Grup çalışmalarını koordine etmek için dijital platformları kullanırım.	Selected	Not Retained
PDCC8	I use digital tools to communicate with peers professionally.	Meslektaşlarımla mesleki iletişim kurmak için dijital araçları kullanırım.	Selected	Retained
PDCC9	I use email or messaging platforms professionally.	E-posta veya mesajlaşma platformlarını mesleki amaçlarla kullanırım.	Rejected	
PDCC10	I respect privacy and confidentiality in digital communication.	Dijital iletişimde mahremiyete ve gizliliğe saygı gösteririm.	Rejected	
PDCC11	I contribute constructively to online academic discussions.	Çevrim içi akademik tartışmalara yapıcı katkıda bulunurum.	Rejected	
PDCC12	I seek professional development opportunities online.	Çevrim içi mesleki gelişim olanakları ararım.	Rejected	
PDCC13	I use digital tools to build professional relationships.	Mesleki ilişkiler kurmak için dijital araçlardan yararlanırım.	Rejected	
PDCC14	I use social media responsibly for professional purposes.	Sosyal medyayı mesleki amaçlarla sorumlu bir şekilde kullanırım.	Rejected	
PDCC15	I stay informed about educational trends through digital networks.	Dijital ağlar aracılığıyla eğitim alanındaki gelişmeleri takip ederim.	Rejected	
AFLA1	I analyze student data from digital systems.	Dijital sistemlerdeki öğrenci verilerini analiz ederim.	Selected	Not Retained
AFLA2	I create online quizzes or assessments.	Çevrim içi kısa sınavlar veya değerlendirmeler hazırlarım.	Selected	Not Retained

AFLA3	I analyze analytics from digital learning platforms to identify student needs.	Öğrencilerin gereksinimlerini belirlemek için dijital öğrenme platformlarındaki analitik verileri incelerim.	Selected	Retained
AFLA4	I implement digital assessment tools to evaluate student learning.	Öğrencilerin öğrenmelerini değerlendirmek için dijital ölçme araçlarını kullanırım.	Selected	Retained
AFLA5	I design formative assessments that leverage digital technologies.	Dijital teknolojilerden yararlanan biçimlendirici değerlendirmeler tasarlarım.	Selected	Retained
AFLA6	I provide feedback through digital platforms.	Dijital platformlar üzerinden geri bildirim veririm.	Selected	Retained
AFLA7	I modify my instructional strategies based on digital assessment evidence.	Dijital değerlendirmelerden elde edilen kanıtlara göre öğretim stratejilerimi değiştiririm.	Selected	Retained
AFLA8	I use digital tools to monitor student progress.	Öğrencilerin ilerlemesini izlemek için dijital araçları kullanırım.	Selected	Retained
AFLA9	I use digital rubrics for grading.	Puanlama yaparken dijital dereceli puanlama anahtarlarını kullanırım.	Rejected	
AFLA10	I incorporate digital portfolios into assessment.	Değerlendirme sürecinde dijital portfolyolardan yararlanırım.	Rejected	
AFLA11	I use digital tools to personalize feedback.	Kişiye özel geri bildirim vermek için dijital araçları kullanırım.	Rejected	
AFLA12	I use technology to support peer assessment.	Akran değerlendirmesini desteklemek için teknolojiden yararlanırım.	Rejected	
AFLA13	I use polling or interactive tools to check understanding.	Öğrencilerin anlayıp anlamadığını belirlemek için anket veya etkileşimli araçlar kullanırım.	Rejected	
AFLA14	I ensure assessments remain valid when delivered digitally.	Dijital ortamda uygulanan değerlendirmelerin geçerliğini korumasını sağlarım.	Rejected	
AFLA15	I design online assignments aligned with objectives.	Öğrenme hedefleriyle uyumlu çevrim içi ödevler tasarlarım.	Rejected	
PATI1	I design learning activities where students must use digital tools to complete meaningful tasks.	Öğrencilerin anlamlı görevleri tamamlamak için dijital araçları kullanmalarını gerektiren öğrenme etkinlikleri tasarlarım.	Selected	Retained
PATI2	I use digital tools to support active learning.	Etkin öğrenmeyi desteklemek için dijital araçları kullanırım.	Selected	Not Retained
PATI3	I ensure technology use supports higher-order thinking.	Teknoloji kullanımının üst düzey düşünmeyi desteklemesini sağlarım.	Selected	Retained
PATI4	I design lessons in which technology is aligned with specific learning objectives.	Teknolojinin belirli öğrenme hedefleriyle uyumlu olduğu dersler tasarlarım.	Selected	Retained
PATI5	I integrate technology to enhance student understanding.	Öğrencilerin konuyu daha iyi anlamalarını sağlamak için teknolojiyi öğretime entegre ederim.	Selected	Not Retained
PATI6	I can choose digital tools that best support specific learning goals.	Belirli öğrenme hedeflerini en iyi destekleyen dijital araçları seçebilirim.	Selected	Retained
PATI7	I ensure that technology use aligns with broader curriculum standards.	Teknoloji kullanımının genel öğretim programı standartlarıyla uyumlu olmasını sağlarım.	Selected	Retained
PATI8	I plan technology use intentionally rather than incidentally.	Teknoloji kullanımını rastlantısal değil, bilinçli bir şekilde planlarım.	Selected	Not Retained
PATI9	I use simulations or interactive tools to explain concepts.	Kavramları açıklamak için simülasyonlardan veya etkileşimli araçlardan yararlanırım.	Rejected	
PATI10	I integrate subject-specific software into teaching.	Alana özgü yazılımları öğretime entegre ederim.	Rejected	
PATI11	I choose technology that supports differentiated instruction.	Farklılaştırılmış öğretimi destekleyen teknolojileri seçerim.	Rejected	
PATI12	I integrate technology to increase student engagement.	Öğrencilerin derse katılımını artırmak için teknolojiyi öğretime entegre ederim.	Rejected	
PATI13	I evaluate the instructional value of digital tools.	Dijital araçların öğretimsel değerini değerlendiririm.	Rejected	
PATI14	I integrate multimedia resources into lesson plans.	Çoklu ortam kaynaklarını ders planlarına entegre ederim.	Rejected	
PATI15	I adapt digital tools to meet instructional goals.	Dijital araçları öğretim hedeflerine uygun hâle getiririm.	Rejected	
ITTA1	I evaluate whether a new digital tool is worth using.	Yeni bir dijital aracın kullanılmaya değer olup olmadığını değerlendiririm.	Selected	Not Retained
ITTA2	I can independently resolve common technical issues during instruction.	Öğretim sırasında karşılaşılan yaygın teknik sorunları bağımsız olarak çözebilirim.	Selected	Retained
ITTA3	I adjust my teaching plans when technology malfunctions.	Teknoloji arızalandığında öğretim planlarımı duruma göre düzenlerim.	Selected	Retained

ITTA4	I remain calm when technology fails during instruction.	Öğretim sırasında teknoloji çalışmadığında sakinliğimi korurum.	Selected	Not Retained
ITTA5	I adapt quickly when digital tools change or update.	Dijital araçlar değiştiğinde veya güncellendiğinde hızla uyum sağlarım.	Selected	Not Retained
ITTA6	I know how to locate reliable digital resources to solve technical problems.	Teknik sorunları çözmek için güvenilir dijital kaynakları nasıl bulacağımı bilirim.	Selected	Retained
ITTA7	I can quickly switch to alternative digital tools when needed.	Gerektiğinde başka dijital araçlara hızla geçebilirim.	Selected	Retained
ITTA8	I can independently learn to use unfamiliar digital tools.	Bilmediğim dijital araçları kullanmayı bağımsız olarak öğrenebilirim.	Selected	Retained
ITTA9	I can independently update my digital skills when necessary.	Gerektiğinde dijital becerilerimi bağımsız olarak geliştirebilirim.	Rejected	
ITTA10	I can diagnose the source of simple technical issues.	Basit teknik sorunların kaynağını belirleyebilirim.	Rejected	
ITTA11	I can resolve common connectivity issues (e.g., Wi-Fi problems).	Yaygın bağlantı sorunlarını (ör. Wi-Fi sorunları) çözebilirim.	Rejected	
ITTA12	I try different strategies when a digital tool does not work as expected.	Bir dijital araç beklendiği gibi çalışmadığında farklı çözüm yolları denerim.	Rejected	
ITTA13	I feel comfortable exploring advanced features of digital tools.	Dijital araçların gelişmiş özelliklerini keşfederken kendimi rahat hissederim.	Rejected	
ITTA14	I seek efficient technological solutions to classroom challenges.	Sınıfta karşılaşılan sorunlara etkili teknolojik çözümler ararım.	Rejected	
ITTA15	I experiment with unfamiliar technologies to understand them.	Bilmediğim teknolojileri anlamak için onları deneyerek keşfederim.	Rejected	
TTSE1	I am confident in learning to use new digital tools independently.	Yeni dijital araçları kullanmayı bağımsız olarak öğrenebileceğime güvenirim.	Selected	Retained
TTSE2	I feel capable of solving technology-related teaching challenges.	Teknolojiyle ilgili öğretim sorunlarını çözebileceğimi hissederim.	Selected	Retained
TTSE3	I believe I can handle unexpected technical problems during a live lesson.	Ders sırasında beklenmedik teknik sorunlarla başa çıkabileceğime inanırım.	Selected	Retained
TTSE4	I believe I can use technology effectively to improve student learning.	Öğrencilerin öğrenmesini geliştirmek için teknolojiyi etkili bir şekilde kullanabileceğime inanırım.	Selected	Retained
TTSE5	I believe I can design lessons that meaningfully incorporate technology.	Teknolojiyi anlamlı bir şekilde içeren dersler tasarlayabileceğime inanırım.	Selected	Not Retained
TTSE6	I feel confident using unfamiliar digital tools during instruction.	Öğretim sırasında bilmediğim dijital araçları kullanma konusunda kendime güvenirim.	Selected	Retained
TTSE7	I feel prepared to teach in a technology-rich environment.	Teknolojinin yoğun olarak kullanıldığı bir ortamda öğretim yapmaya hazır olduğumu hissederim.	Selected	Not Retained
TTSE8	I am confident adapting instruction using digital tools.	Dijital araçları kullanarak öğretimi uyarlama konusunda kendime güvenirim.	Selected	Not Retained
TTSE9	I feel competent using digital assessment tools.	Dijital değerlendirme araçlarını kullanma konusunda kendimi yeterli hissederim.	Rejected	
TTSE10	I believe I can keep up with technological changes in education.	Eğitim alanındaki teknolojik değişimlere ayak uydurabileceğime inanırım.	Rejected	
TTSE11	I feel confident guiding students in using technology.	Öğrencilere teknoloji kullanımı konusunda rehberlik ederken kendime güvenirim.	Rejected	
TTSE12	I trust my ability to select appropriate digital tools.	Uygun dijital araçları seçme becerime güvenirim.	Rejected	
TTSE13	I feel capable of integrating technology across subjects.	Teknolojiyi farklı ders alanlarına entegre edebileceğimi hissederim.	Rejected	
TTSE14	I believe I can use technology to enhance learning outcomes.	Öğrenme çıktılarını geliştirmek için teknolojiyi kullanabileceğime inanırım.	Rejected	
TTSE15	I am confident experimenting with new digital teaching strategies.	Yeni dijital öğretim stratejilerini deneme konusunda kendime güvenirim.	Rejected	

Appendix B. Item-Level Content-Validity Ratings and Retention Decisions

Item	Auth1	Auth2	Exp1	Exp2	Exp3	Exp4	Exp5	Exp6	Exp7	Avg Scr	I-CVI	S-CVI/Ave (All)	S-CVI/Ave (Selected)	Status	After EFA
FDTU1	3	4	3	3	3	3	3	4	4	3.33	1.00			Selected	Retained
FDTU2	4	3	3	3	4	3	4	3	3	3.33	1.00			Selected	Retained
FDTU3	4	4	4	3	3	2	4	3	3	3.33	0.89			Selected	Retained
FDTU4	3	3	3	3	3	3	3	4	4	3.22	1.00			Selected	Retained
FDTU5	4	4	2	3	4	2	3	3	3	3.11	0.78			Selected	Retained
FDTU6	3	3	2	3	3	4	3	2	3	2.89	0.78			Selected	Retained
FDTU7	3	3	2	3	4	3	3	2	4	3.00	0.78			Selected	Not Retained
FDTU8	4	4	2	4	3	2	3	4	4	3.33	0.78	0.63	0.88	Selected	Not Retained
FDTU9	2	2	2	2	3	3	2	2	3	2.33	0.33			Rejected	
FDTU10	1	2	1	1	3	2	3	3	3	2.11	0.44			Rejected	
FDTU11	1	3	2	3	2	1	3	3	1	2.11	0.44			Rejected	
FDTU12	3	1	1	3	1	2	3	1	3	2.00	0.44			Rejected	
FDTU13	1	1	2	3	1	3	2	2	3	2.00	0.33			Rejected	
FDTU14	1	3	2	1	2	1	3	3	1	1.89	0.33			Rejected	
FDTU15	1	2	2	3	1	2	1	2	1	1.67	0.11			Rejected	
AFLA1	4	3	3	3	4	4	4	2	4	3.44	0.89			Selected	Not Retained
AFLA2	3	4	3	4	3	2	4	4	4	3.44	0.89			Selected	Not Retained
AFLA3	4	3	3	4	3	2	3	4	4	3.33	0.89			Selected	Retained
AFLA4	3	4	3	3	4	3	3	4	3	3.33	1.00			Selected	Retained
AFLA5	3	4	3	3	3	3	4	3	4	3.33	1.00			Selected	Retained
AFLA6	4	3	2	4	4	2	4	3	3	3.22	0.78			Selected	Retained
AFLA7	3	3	2	3	4	4	4	3	3	3.22	0.89			Selected	Retained
AFLA8	4	4	2	3	4	3	3	2	3	3.11	0.78	0.59	0.89	Selected	Retained
AFLA9	3	1	3	3	3	2	3	1	2	2.33	0.56			Rejected	
AFLA10	2	2	3	2	1	2	3	2	3	2.22	0.33			Rejected	
AFLA11	1	2	1	2	2	2	3	1	1	1.67	0.11			Rejected	
AFLA12	3	2	1	1	1	2	3	2	2	1.89	0.22			Rejected	
AFLA13	1	2	3	1	1	3	2	2	2	1.89	0.22			Rejected	
AFLA14	1	2	1	2	2	2	1	1	3	1.67	0.11			Rejected	
AFLA15	2	2	1	1	1	3	1	1	1	1.44	0.11			Rejected	
ITTA1	4	3	3	4	4	3	3	3	3	3.33	1.00			Selected	Not Retained
ITTA2	4	4	4	4	3	4	4	2	4	3.67	0.89			Selected	Retained
ITTA3	4	4	4	4	4	2	4	3	4	3.67	0.89			Selected	Retained
ITTA4	3	4	2	4	3	2	4	3	4	3.22	0.78			Selected	Not Retained
ITTA5	4	3	3	3	3	3	3	4	3	3.22	1.00	0.64	0.93	Selected	Not Retained
ITTA6	4	3	3	4	4	3	4	4	3	3.56	1.00			Selected	Retained
ITTA7	4	4	4	3	3	4	4	2	4	3.56	0.89			Selected	Retained
ITTA8	3	3	4	3	3	3	4	3	4	3.33	1.00			Selected	Retained
ITTA9	1	2	3	2	2	3	3	2	2	2.22	0.33			Rejected	
ITTA10	3	1	3	2	3	1	3	2	2	2.22	0.44			Rejected	

ITTA11	3	1	2	2	3	3	1	2	3	2.22	0.44			Rejected	
ITTA12	3	2	3	3	1	1	2	1	2	2.00	0.33			Rejected	
ITTA13	2	2	2	2	1	2	3	1	3	2.00	0.22			Rejected	
ITTA14	2	3	2	1	2	1	3	2	1	1.89	0.22			Rejected	
ITTA15	1	1	3	2	1	1	1	3	1	1.56	0.22			Rejected	
PATI1	4	3	4	4	3	3	3	3	4	3.44	1.00			Selected	Retained
PATI2	3	4	3	4	4	3	4	2	4	3.44	0.89			Selected	Not Retained
PATI3	4	3	4	4	3	3	4	2	3	3.33	0.89			Selected	Retained
PATI4	3	4	4	4	3	3	3	2	4	3.33	0.89			Selected	Retained
PATI5	3	4	4	4	3	4	3	2	3	3.33	0.89			Selected	Not Retained
PATI6	4	4	2	4	3	2	3	3	3	3.11	0.78			Selected	Retained
PATI7	3	3	2	3	4	2	4	3	4	3.11	0.78			Selected	Retained
PATI8	3	4	2	4	3	4	3	3	3	3.22	0.89	0.61	0.88	Selected	Not Retained
PATI9	2	1	3	2	3	2	2	2	3	2.22	0.33			Rejected	
PATI10	3	2	3	3	2	3	1	1	2	2.22	0.44			Rejected	
PATI11	2	3	1	1	2	2	3	3	2	2.11	0.33			Rejected	
PATI12	3	3	2	1	1	2	3	2	2	2.11	0.33			Rejected	
PATI13	3	2	1	1	2	3	1	2	2	1.89	0.22			Rejected	
PATI14	3	3	1	2	2	1	1	3	1	1.89	0.33			Rejected	
PATI15	1	2	2	3	2	2	1	1	2	1.78	0.11			Rejected	
TTSE1	4	4	3	4	4	4	4	2	3	3.56	0.89			Selected	Retained
TTSE2	3	3	4	3	4	3	3	4	3	3.33	1.00			Selected	Retained
TTSE3	3	3	3	3	4	4	4	2	4	3.33	0.89			Selected	Retained
TTSE4	4	3	4	4	3	3	3	2	3	3.22	0.89			Selected	Retained
TTSE5	4	4	4	3	3	4	4	4	4	3.78	1.00			Selected	Not Retained
TTSE6	3	4	4	4	3	3	3	2	3	3.22	0.89			Selected	Retained
TTSE7	3	4	2	3	3	3	3	3	4	3.11	0.89			Selected	Not Retained
TTSE8	3	3	2	4	3	3	3	2	4	3.00	0.78	0.61	0.90	Selected	Not Retained
TTSE9	3	3	2	1	2	3	3	1	1	2.11	0.44			Rejected	
TTSE10	2	2	3	3	3	1	1	3	1	2.11	0.44			Rejected	
TTSE11	3	2	2	1	1	3	2	3	2	2.11	0.33			Rejected	
TTSE12	1	3	2	2	2	2	2	2	1	1.89	0.11			Rejected	
TTSE13	1	3	1	3	1	2	1	2	1	1.67	0.22			Rejected	
TTSE14	3	1	2	1	1	1	2	1	3	1.67	0.22			Rejected	
TTSE15	1	1	1	2	1	1	1	1	3	1.33	0.11			Rejected	
PDCC1	4	4	3	4	3	3	4	4	3	3.56	1.00			Selected	Retained
PDCC2	4	3	3	3	4	3	4	4	4	3.56	1.00			Selected	Retained
PDCC3	4	4	3	4	4	4	3	2	3	3.44	0.89	0.67	0.90	Selected	Retained
PDCC4	3	4	2	3	3	4	3	2	4	3.11	0.78			Selected	Not Retained
PDCC5	4	3	4	3	3	4	3	3	4	3.44	1.00			Selected	Retained
PDCC6	4	4	2	3	3	4	3	4	4	3.44	0.89			Selected	Retained

PDCC7	3	3	2	4	4	3	4	2	4	3.22	0.78	Selected	Not Retained
PDCC8	3	3	4	4	4	4	3	2	3	3.33	0.89	Selected	Retained
PDCC9	3	3	2	3	3	1	2	2	3	2.44	0.56	Rejected	
PDCC10	2	3	3	1	2	3	2	3	1	2.22	0.44	Rejected	
PDCC11	3	2	3	2	1	3	3	2	1	2.22	0.44	Rejected	
PDCC12	3	2	2	2	3	2	2	1	2	2.11	0.22	Rejected	
PDCC13	3	3	3	2	3	1	1	2	1	2.11	0.44	Rejected	
PDCC14	2	3	1	2	1	1	3	3	3	2.11	0.44	Rejected	
PDCC15	2	3	1	1	1	1	2	3	2	1.78	0.22	Rejected	

Appendix C. Bilingual 18-Item Scenario-Based Quiz and Answer Key

Construct	Quiz Item	Field	English	Turkish	Correct Answer
FDTU	1	Scenario	A pre-service teacher must upload a lesson plan, presentation, and video to the learning management system. The video exceeds the upload limit.	Bir öğretmen adayı ders planını, sunusunu ve videosunu öğrenme yönetim sistemine yüklemelidir. Video, yükleme boyutu sınırını aşmaktadır.	
		Question	What is the most appropriate action?	En uygun çözüm hangisidir?	
		Answer Choice 1	Email all files separately and ignore the required submission area.	Dosyaların tümünü ayrı ayrı e-postayla gönderip zorunlu teslim alanını kullanmamak.	
		Answer Choice 2	Delete parts of the lesson video without checking their instructional importance.	Öğretim açısından önemli olup olmadığını değerlendirmeden videonun bazı bölümlerini silmek.	
		Answer Choice 3	Compress the video or upload it to an approved cloud service, check access permissions, and submit the link with the other files.	Videoyu sıkıştırmak veya onaylı bir bulut hizmetine yüklemek, erişim izinlerini kontrol etmek ve bağlantıyı diğer dosyalarla birlikte teslim etmek.	Correct
		Answer Choice 4	Rename the video with a shorter filename and try repeatedly.	Videonun dosya adını kısaltıp tekrar tekrar yüklemeyi denemek.	
		Answer Choice 5	Upload only the lesson plan because it is the main document.	Temel belge olduğu için yalnızca ders planını yüklemek.	
	2	Scenario	During a microteaching session, a pre-service teacher must display a presentation from a laptop on the classroom projector, but no image appears.	Bir öğretmen adayı mikro öğretim sırasında dizüstü bilgisayarındaki sunuyu sınıftaki projeksiyon cihazında göstermelidir, ancak ekranda görüntü oluşmaz.	
		Question	What should the teacher do first?	Öğretmen adayı önce ne yapmalıdır?	
		Answer Choice 1	Immediately cancel the technology-supported part of the lesson.	Dersin teknoloji destekli bölümünü hemen iptal etmek.	
		Answer Choice 2	Check the cable or wireless connection, select the correct display input, and verify the laptop display settings.	Kabloyu veya kablosuz bağlantıyı kontrol etmek, doğru görüntü girişini seçmek ve bilgisayarın ekran ayarlarını doğrulamak.	Correct
		Answer Choice 3	Restart every classroom device before checking any connection.	Bağlantıları kontrol etmeden sınıftaki bütün cihazları yeniden başlatmak.	
		Answer Choice 4	Ask students to solve the problem while the lesson stops.	Dersi durdurup sorunu öğrencilerin çözmesini istemek.	
		Answer Choice 5	Continue speaking and assume the image will eventually appear.	Konuşmaya devam edip görüntünün bir süre sonra kendiliğinden geleceğini varsaymak.	
	3	Scenario	A pre-service teacher and four peers are jointly preparing a unit plan. Different versions of the same document are circulating by email, and recent changes are being lost.	Bir öğretmen adayı ve dört arkadaşı birlikte bir ünite planı hazırlamaktadır. Aynı belgenin farklı sürümleri e-postayla dolaşmakta ve son değişiklikler kaybolmaktadır.	
		Question	Which solution best addresses the problem?	Sorunu en iyi çözen uygulama hangisidir?	
		Answer Choice 1	Ask one person to rewrite the plan from memory.	Bir kişiden planı hatırladığı kadarıyla yeniden yazmasını istemek.	

		Answer Choice 2	Continue exchanging files but add the date to each filename.	Dosya alışverişini sürdürüp her dosya adına tarih eklemek.	
		Answer Choice 3	Print every version and compare them manually.	Bütün sürümleri yazdırıp elle karşılaştırmak.	
		Answer Choice 4	Use a shared document with editing permissions and version history, and agree on file-management rules.	Düzenleme izinleri ve sürüm geçmişi bulunan ortak bir belge kullanmak ve dosya yönetimi kuralları üzerinde anlaşmak.	Correct
		Answer Choice 5	Allow each group member to submit a different unit plan.	Her grup üyesinin farklı bir ünite planı teslim etmesine izin vermek.	
PDCC	1	Scenario	A pre-service teacher needs to send practicum feedback containing student performance information to a mentor teacher.	Bir öğretmen adayının öğrenci performans bilgilerini içeren uygulama geri bildirimini danışman öğretmene göndermesi gerekir.	
		Question	Which digital communication practice is most appropriate?	En uygun dijital iletişim uygulaması hangisidir?	
		Answer Choice 1	Use the institution-approved platform, share only necessary information with the authorized mentor, and verify the recipient before sending.	Kurum tarafından onaylanan platformu kullanmak, yalnızca gerekli bilgileri yetkili danışmanla paylaşmak ve göndermeden önce alıcıyı doğrulamak.	Correct
		Answer Choice 2	Post the document in the class messaging group so the mentor sees it quickly.	Danışmanın hızlıca görmesi için belgeyi sınıf mesajlaşma grubunda paylaşmak.	
		Answer Choice 3	Send it through a personal social-media account because it is convenient.	Kolay olduğu için kişisel bir sosyal medya hesabından göndermek.	
		Answer Choice 4	Remove the filename but leave all student identifiers in the document.	Dosya adını kaldırıp belgedeki öğrenci kimlik bilgilerini olduğu gibi bırakmak.	
		Answer Choice 5	Upload it publicly and send the mentor the link.	Belgeyi herkese açık olarak yükleyip bağlantıyı danışmana göndermek.	
	2	Scenario	Members of an online lesson-design group disagree about which digital tool to use. The discussion has become repetitive and several members no longer contribute.	Çevrim içi ders tasarımı grubunun üyeleri hangi dijital aracın kullanılacağı konusunda anlaşamaz. Tartışma tekrara dönüşür ve bazı üyeler artık katkıda bulunmaz.	
		Question	What is the best collaborative response?	İş birliğini en iyi destekleyen yaklaşım hangisidir?	
		Answer Choice 1	Choose the tool preferred by the most active member without discussion.	Tartışmadan en etkin üyenin tercih ettiği aracı seçmek.	
		Answer Choice 2	Create a second group that excludes members who disagree.	Farklı düşünen üyeleri dışarıda bırakan ikinci bir grup kurmak.	
		Answer Choice 3	Continue debating until everyone independently reaches the same preference.	Herkes aynı tercihe ulaşıncaya kadar tartışmayı sürdürmek.	
		Answer Choice 4	Ask the instructor to complete the group decision.	Grup kararını öğretim üyesinin vermesini istemek.	
		Answer Choice 5	Summarize the alternatives, agree on instructional criteria, invite each member's input, and document the decision.	Seçenekleri özetlemek, öğretimsel ölçütler üzerinde uzlaşmak, her üyenin görüşünü almak ve kararı kaydetmek.	Correct
	3	Scenario	A pre-service teacher finds an online community where teachers exchange lesson materials. One resource looks useful but its author and usage rights are unclear.	Bir öğretmen adayı, öğretmenlerin ders materyali paylaştığı çevrim içi bir topluluk bulur. Bir kaynak yararlı görünmektedir, ancak yazarı ve kullanım hakları belirsizdir.	
		Question	What should the teacher do?	Öğretmen adayı ne yapmalıdır?	
		Answer Choice 1	Download it, remove its identifying information, and present it as original work.	Kaynağı indirip kimlik bilgilerini kaldırmak ve özgün çalışma olarak sunmak.	
		Answer Choice 2	Share it widely because it was available online.	Çevrim içi erişilebildiği için kaynağı geniş bir grupla paylaşmak.	
		Answer Choice 3	Verify the source and licence, request permission when necessary, and acknowledge the creator when using it.	Kaynağı ve kullanım lisansını doğrulamak, gerektiğinde izin istemek ve kullanırken hazırlayan kişiyi belirtmek.	Correct
		Answer Choice 4	Assume educational materials never require attribution.	Eğitim materyallerinde hiçbir zaman kaynak belirtmek gerekmediğini varsaymak.	
		Answer Choice 5	Avoid all resources created by other teachers.	Diğer öğretmenlerin hazırladığı bütün kaynaklardan kaçınmak.	

AFLA	1	Scenario	After an online quiz, a teacher sees that most students selected the same incorrect option on one learning objective.	Çevrim içi bir kısa sınavdan sonra öğretmen, öğrencilerin çoğunun belirli bir öğrenme hedefindeki aynı yanlış seçeneği işaretlediğini görür.	
		Question	What is the best instructional use of this information?	Bu bilgi öğretimde en uygun nasıl kullanılmalıdır?	
		Answer Choice 1	Examine the item and response pattern, identify the likely misconception, reteach the concept, and check understanding again.	Maddeyi ve cevap örüntüsünü incelemek, olası kavram yanılgısını belirlemek, konuyu yeniden öğretmek ve anlayışı tekrar kontrol etmek.	Correct
		Answer Choice 2	Lower every student's grade because the error was common.	Hata yaygın olduğu için bütün öğrencilerin notunu düşürmek.	
		Answer Choice 3	Delete the objective from the course.	Öğrenme hedefini dersten çıkarmak.	
		Answer Choice 4	Assume students did not study and continue to the next topic.	Öğrencilerin çalışmadığını varsayıp sonraki konuya geçmek.	
		Answer Choice 5	Repeat the same quiz without providing instruction or feedback.	Öğretim veya geri bildirim sunmadan aynı sınavı tekrarlamak.	
	2	Scenario	A digital quiz reports that one student completed all items unusually quickly and obtained a perfect score, although previous work showed substantial difficulty.	Dijital bir kısa sınavda, önceki çalışmalarında önemli güçlük yaşayan bir öğrencinin bütün maddeleri olağan dışı bir hızla tamamlayıp tam puan aldığı görülür.	
		Question	What is the most appropriate interpretation?	En uygun yorum hangisidir?	
		Answer Choice 1	Conclude immediately that the student cheated.	Öğrencinin kopya çektiği sonucuna hemen varmak.	
		Answer Choice 2	Accept the score as definitive proof of mastery.	Puanı tam öğrenmenin kesin kanıtı olarak kabul etmek.	
		Answer Choice 3	Discard the score because completion time was short.	Tamamlama süresi kısa olduğu için puanı geçersiz saymak.	
		Answer Choice 4	Treat the pattern as a signal for further evidence, review item-level data, and use a follow-up assessment or conversation.	Örüntüyü ek kanıt gerektiren bir işaret olarak görmek, madde düzeyindeki verileri incelemek ve izleme değerlendirmesi veya görüşme yapmak.	Correct
		Answer Choice 5	Replace all future quizzes with oral examinations.	Bundan sonraki bütün kısa sınavları sözlü sınavla değiştirmek.	
	3	Scenario	Students submit digital concept maps. The teacher wants feedback to be timely, consistent, and linked to the stated learning criteria.	Öğrenciler dijital kavram haritaları teslim eder. Öğretmen geri bildirimin zamanında, tutarlı ve açıklanan öğrenme ölçütleriyle bağlantılı olmasını ister.	
		Question	Which approach is best?	En uygun yaklaşım hangisidir?	
		Answer Choice 1	Send every student the same general comment.	Her öğrenciye aynı genel yorumu göndermek.	
		Answer Choice 2	Use only an automatically generated numerical score.	Yalnızca otomatik olarak oluşturulan sayısal puanı kullanmak.	
		Answer Choice 3	Comment only on visual attractiveness.	Yalnızca görsel çekicilik hakkında yorum yapmak.	
		Answer Choice 4	Wait until the end of the term and provide one combined comment.	Dönem sonuna kadar bekleyip tek bir toplu yorum vermek.	
		Answer Choice 5	Use a digital rubric aligned with the criteria and add specific comments identifying strengths and next steps.	Ölçütlerle uyumlu dijital bir dereceli puanlama anahtarı kullanmak ve güçlü yönlerle sonraki adımları belirten özel yorumlar eklemek.	Correct
PATI	1	Scenario	A teacher is selecting technology for a lesson whose objective is for students to compare competing explanations and defend a conclusion with evidence.	Bir öğretmen, öğrencilerin farklı açıklamaları karşılaştırıp kanıta dayalı bir sonucu savunmalarını amaçlayan ders için teknoloji seçmektedir.	
		Question	Which choice is most pedagogically aligned?	Hangi seçim pedagojik açıdan en uyumludur?	
		Answer Choice 1	A slideshow that presents the teacher's conclusion for students to copy.	Öğretmenin sonucunu öğrencilerin kopyalaması için sunan bir slayt gösterisi.	
		Answer Choice 2	A collaborative evidence-mapping tool in which students classify evidence, compare explanations, and justify conclusions.	Öğrencilerin kanıtları sınıflandırdığı, açıklamaları karşılaştırdığı ve sonuçlarını gerekçelendirdiği ortak bir kanıt haritalama aracı.	Correct
		Answer Choice 3	An animation unrelated to the competing explanations.	Farklı açıklamalarla ilgisi olmayan bir animasyon.	
		Answer Choice 4	A timed multiple-choice drill focused only on recalling definitions.	Yalnızca tanımları hatırlamaya odaklanan süreli çoktan seçmeli alıştırma.	

		Answer Choice 5	The newest available application, regardless of its functions.	İşlevlerinden bağımsız olarak mevcut en yeni uygulama.	
	2	Scenario	A teacher plans to use virtual reality in a geography lesson, but the learning objective is simply to identify five map symbols. Setup will take half of the lesson.	Bir öğretmen coğrafya dersinde sanal gerçeklik kullanmayı planlar, ancak öğrenme hedefi yalnızca beş harita sembolünü tanımaktır. Kurulum dersin yarısını alacaktır.	
		Question	What is the best decision?	En uygun karar hangisidir?	
		Answer Choice 1	Use a simpler tool or non-digital activity that efficiently addresses the objective, reserving virtual reality for a task that benefits from immersion.	Hedefi verimli biçimde karşılayan daha basit bir araç veya dijital olmayan etkinlik kullanmak; sanal gerçekliği, sürükleyici deneyimden yararlanacak bir görev için saklamak.	Correct
		Answer Choice 2	Use virtual reality because advanced technology always improves learning.	İleri teknoloji öğrenmeyi her zaman geliştirdiği için sanal gerçekliği kullanmak.	
		Answer Choice 3	Change the objective after the lesson so it matches the tool.	Dersten sonra öğrenme hedefini araca uyacak şekilde değiştirmek.	
		Answer Choice 4	Use virtual reality but omit any assessment of learning.	Sanal gerçekliği kullanıp öğrenmeyi değerlendirmemek.	
		Answer Choice 5	Ask students to explore freely without explaining the objective.	Hedefi açıklamadan öğrencilerin serbestçe keşfetmesini istemek.	
	3	Scenario	Students in a science class must understand how changing one variable affects a system over time. A static diagram has not made the relationship clear.	Fen dersindeki öğrencilerin bir değişkendeki değişimin sistemi zaman içinde nasıl etkilediğini anlamaları gerekir. Sabit bir şema ilişkiyi açıklamakta yetersiz kalmıştır.	
		Question	Which technology use best supports the objective?	Hangi teknoloji kullanımı hedefi en iyi destekler?	
		Answer Choice 1	Show a decorative video that contains no controllable variables.	Kontrol edilebilir değişken içermeyen görsel açıdan çekici bir video göstermek.	
		Answer Choice 2	Ask students to copy definitions into a word-processing document.	Öğrencilerden tanımları bir kelime işlemci belgesine kopyalamalarını istemek.	
		Answer Choice 3	Use an interactive simulation in which students manipulate the variable, record outcomes, and explain the observed pattern.	Öğrencilerin değişkeni değiştirdiği, sonuçları kaydettiği ve gözlenen örüntüyü açıkladığı etkileşimli bir simülasyon kullanmak.	Correct
		Answer Choice 4	Replace the science objective with practice in using the simulation interface.	Fen öğrenme hedefini simülasyon arayüzünü kullanma alıştırmasıyla değiştirmek.	
		Answer Choice 5	Let students use any application without a common task.	Ortak bir görev vermeden öğrencilerin istedikleri uygulamayı kullanmasına izin vermek.	
ITTA	1	Scenario	Five minutes before a technology-supported lesson, the classroom internet connection fails. The activity requires students to examine online primary sources.	Teknoloji destekli bir dersten beş dakika önce sınıfın internet bağlantısı kesilir. Etkinlikte öğrencilerin çevrim içi birincil kaynakları incelemesi gerekmektedir.	
		Question	What is the best response?	En uygun çözüm hangisidir?	
		Answer Choice 1	Cancel the learning objective and allow free time.	Öğrenme hedefini iptal edip öğrencilere serbest zaman vermek.	
		Answer Choice 2	Spend the entire lesson repeatedly reconnecting to the same network.	Dersin tamamını aynı ağa tekrar tekrar bağlanmaya çalışarak geçirmek.	
		Answer Choice 3	Lecture on an unrelated topic.	İlgisiz bir konuda düz anlatım yapmak.	
		Answer Choice 4	Tell students the activity is impossible without internet.	İnternet olmadan etkinliğin yapılamayacağını söylemek.	
		Answer Choice 5	Use previously downloaded or printed source sets and continue the same analysis task, then restore online access later.	Önceden indirilmiş veya basılmış kaynak setlerini kullanarak aynı analiz görevini sürdürmek ve çevrim içi erişimi daha sonra yeniden sağlamak.	Correct
	2	Scenario	During group presentations, the classroom audio repeatedly cuts out. The presentation files contain essential student-recorded interviews.	Grup sunumları sırasında sınıftaki ses sistemi sürekli kesilir. Sunu dosyalarında öğrencilerin kaydettiği ve ders için gerekli olan görüşmeler bulunmaktadır.	
		Question	What should the teacher do?	Öğretmen ne yapmalıdır?	
		Answer Choice 1	End all presentations and grade only the slides.	Bütün sunumları bitirip yalnızca slaytları değerlendirmek.	

		Answer Choice 2	Increase the volume repeatedly without checking the cause.	Sorunun kaynağını kontrol etmeden sesi tekrar tekrar yükseltmek.	
		Answer Choice 3	Ask each group to describe the interviews from memory.	Her gruptan görüşmeleri hatırladığı kadarıyla anlatmasını istemek.	
		Answer Choice 4	Check the audio output and connections, then use a prepared alternative device or share the files for individual listening if the fault continues.	Ses çıkışını ve bağlantıları kontrol etmek; sorun sürerse hazır bir yedek cihaz kullanmak veya dosyaları bireysel dinleme için paylaşmak.	Correct
		Answer Choice 5	Ignore the interview evidence and continue as though it played correctly.	Görüşme kanıtlarını görmezden gelip doğru biçimde oynatılmış gibi devam etmek.	
	3	Scenario	A learning application updates overnight and the buttons used in a planned activity have moved. Students are waiting to begin.	Bir öğrenme uygulaması gece güncellenir ve planlanan etkinlikte kullanılacak düğmelerin yerleri değişir. Öğrenciler etkinliğe başlamayı beklemektedir.	
		Question	What is the most adaptive response?	Uyarlanabilirliği en iyi gösteren tepki hangisidir?	
		Answer Choice 1	Abandon the lesson because the interface is unfamiliar.	Arayüz yabancı olduğu için dersten vazgeçmek.	
		Answer Choice 2	Quickly locate the required functions using help or a brief test, give students revised directions, and switch to the prepared alternative if necessary.	Yardım bölümünü veya kısa bir denemeyi kullanarak gerekli işlevleri hızla bulmak, öğrencilere güncellenmiş yönergeler vermek ve gerekirse hazırlanan alternatif araca geçmek.	Correct
		Answer Choice 3	Ask students to explore every new feature before beginning.	Başlamadan önce öğrencilerden bütün yeni özellikleri keşfetmelerini istemek.	
		Answer Choice 4	Continue giving the old directions even though they no longer match the interface.	Artık arayüzle uyuşmadığı hâlde eski yönergeleri vermeyi sürdürmek.	
		Answer Choice 5	Postpone the course indefinitely until the earlier version returns.	Eski sürüm geri gelinceye kadar dersi belirsiz süreyle ertelemek.	
TTSE	1	Scenario	A pre-service teacher has practised with a digital formative-assessment tool but feels nervous about using it during the first practicum lesson.	Bir öğretmen adayı dijital biçimlendirici değerlendirme aracıyla alıştırma yapmıştır, ancak ilk uygulama dersinde bu aracı kullanma konusunda kaygılıdır.	
		Question	Which response best demonstrates strong, realistic technology self-efficacy?	Hangi davranış güçlü ve gerçekçi teknoloji öz yeterliğini en iyi gösterir?	
		Answer Choice 1	Avoid the tool until complete confidence develops without practice.	Uygulama yapmadan tam güven oluşuncaya kadar aracı kullanmamak.	
		Answer Choice 2	Use the tool without preparation because confidence means expecting no difficulty.	Öz güvenin hiç güçlük beklememek anlamına geldiğini düşünerek hazırlıksız kullanmak.	
		Answer Choice 3	Ask the mentor to operate the tool while taking no responsibility.	Hiç sorumluluk almadan aracı danışman öğretmenin kullanmasını istemek.	
		Answer Choice 4	Replace the assessment objective with an easier activity.	Değerlendirme hedefini daha kolay bir etkinlikle değiştirmek.	
		Answer Choice 5	Rehearse the procedure, prepare a backup, use the tool, and treat manageable difficulties as opportunities to improve.	Uygulamayı prova etmek, bir yedek plan hazırlamak, aracı kullanmak ve yönetilebilir güçlükleri gelişme fırsatı olarak görmek.	Correct
	2	Scenario	A pre-service teacher is asked to design a technology-supported lesson using an unfamiliar application.	Bir öğretmen adayından bilmediği bir uygulamayı kullanarak teknoloji destekli bir ders tasarlaması istenir.	
		Question	Which thought and action reflect the strongest self-efficacy?	Hangi düşünce ve davranış en güçlü öz yeterliği yansıtır?	
		Answer Choice 1	“Because I have never used it, I will ask someone else to design the lesson.”	“Daha önce kullanmadığım için dersi başka birinden tasarlamasını isteyeceğim.”	
		Answer Choice 2	“I can learn the essential functions through guided practice, test the lesson, and seek targeted help if a problem remains.”	“Yönlendirilmiş alıştırmayla gerekli işlevleri öğrenebilir, dersi deneyebilir ve sorun kalırsa belirli bir konuda yardım isteyebilirim.”	Correct
		Answer Choice 3	“If the application is useful, no preparation will be necessary.”	“Uygulama yararlıysa hazırlık yapmam gerekmez.”	
		Answer Choice 4	“One initial mistake would prove that I am not capable of technology-supported teaching.”	“İlk denemedeki bir hata, teknoloji destekli öğretimde yetersiz olduğumu kanıtlar.”	

	Answer Choice 5	"I will use the application only if a peer guarantees that nothing will go wrong."	"Yalnızca bir arkadaşım hiçbir sorun çıkmayacağını garanti ederse uygulamayı kullanacağım."	
3	Scenario	During a practicum lesson, a digital activity does not proceed exactly as planned. The learning objective is still achievable, and students remain engaged.	Bir uygulama dersinde dijital etkinlik tam olarak planlandığı gibi ilerlemez. Öğrenme hedefine hâlâ ulaşılabilir ve öğrenciler derse katılmayı sürdürmektedir.	
	Question	Which response best reflects teaching-related technology self-efficacy?	Hangi davranış öğretimle ilgili teknoloji öz yeterliğini en iyi yansıtır?	
	Answer Choice 1	Conclude that technology should never be used again.	Teknolojinin bir daha asla kullanılmaması gerektiği sonucuna varmak.	
	Answer Choice 2	Pretend no problem occurred and make no adjustment.	Hiç sorun olmamış gibi davranıp hiçbir düzenleme yapmamak.	
	Answer Choice 3	Make a purposeful adjustment, continue toward the learning objective, and later reflect on how to improve the activity.	Amaca uygun bir düzenleme yapmak, öğrenme hedefine doğru ilerlemeyi sürdürmek ve daha sonra etkinliğin nasıl geliştirileceğini değerlendirmek.	Correct
	Answer Choice 4	Hand control of the lesson to a student immediately.	Dersin yönetimini hemen bir öğrenciye bırakmak.	
	Answer Choice 5	Stop the lesson to protect confidence from further difficulty.	Başka bir güçlük yaşamamak ve öz güveni korumak için dersi durdurmak.	

Appendix D. Pattern Matrix for the Final 33-Item Exploratory Factor Solution

Item	Factor 1	Factor 2	Factor 3	Factor 4	Factor 5	Factor 6
PDCC01	-0.034	**0.796**	0.019	-0.010	0.041	0.008
PDCC02	0.041	**0.723**	-0.046	0.007	-0.028	0.146
PDCC03	-0.075	**0.803**	-0.007	-0.005	0.040	0.011
PDCC05	0.011	**0.756**	-0.035	0.037	-0.042	-0.044
PDCC06	0.014	**0.800**	0.058	-0.045	0.019	-0.042
PDCC08	0.016	**0.790**	0.013	0.025	-0.017	-0.064
AFLA03	0.008	0.013	**0.777**	-0.040	0.019	0.006
AFLA04	-0.028	0.017	**0.821**	-0.053	0.007	-0.005
AFLA05	0.012	-0.009	**0.675**	0.160	-0.023	0.011
AFLA06	-0.064	-0.052	**0.799**	-0.006	-0.007	-0.011
AFLA07	0.038	0.082	**0.752**	-0.006	-0.023	0.016
AFLA08	0.033	-0.049	**0.820**	-0.029	0.026	-0.009
TTSE01	0.079	0.088	-0.004	-0.028	0.024	**0.733**
TTSE02	-0.076	-0.067	-0.008	0.020	0.056	**0.703**
TTSE03	-0.014	-0.007	-0.082	0.041	-0.021	**0.812**
TTSE04	-0.010	-0.009	0.043	-0.076	-0.002	**0.760**
TTSE06	0.036	0.001	0.055	0.041	-0.057	**0.704**
ITTA02	-0.007	0.004	-0.009	0.031	**0.797**	-0.027
ITTA03	0.062	-0.003	-0.033	-0.014	**0.797**	0.016
ITTA06	0.056	0.009	0.032	-0.006	**0.801**	-0.053
ITTA07	-0.071	-0.009	0.008	-0.007	**0.842**	0.068
ITTA08	-0.022	0.010	0.005	0.013	**0.804**	0.001
PATI01	0.001	-0.004	-0.083	**0.832**	-0.014	0.018

PATI03	-0.016	0.013	0.014	**0.797**	0.054	-0.030
PATI04	0.006	-0.002	0.061	**0.841**	-0.017	-0.005
PATI06	-0.028	0.027	0.035	**0.813**	-0.054	0.006
PATI07	0.037	-0.017	-0.020	**0.803**	0.046	0.007
FDTU01	**0.768**	0.036	-0.041	0.025	0.000	-0.028
FDTU02	**0.789**	0.043	-0.017	-0.080	0.007	-0.051
FDTU03	**0.795**	-0.020	0.019	0.019	0.002	-0.023
FDTU04	**0.847**	-0.065	0.050	0.000	0.022	0.031
FDTU05	**0.804**	0.033	0.009	-0.016	-0.014	0.062
FDTU06	**0.794**	-0.058	-0.016	0.050	-0.007	0.008

Note. Principal axis factoring with Promax rotation was applied. The table reports pattern coefficients for the final 33-item solution. Factor 1 = Foundational Digital Tool Use; Factor 2 = Professional Digital Communication and Collaboration; Factor 3 = Assessment, Feedback, and Learning Analytics; Factor 4 = Pedagogically Aligned Technology Integration; Factor 5 = Instructional Technology Troubleshooting and Adaptability; Factor 6 = Teaching-Related Technology Self-Efficacy.
